# Origin of volatile organic compound emissions from subarctic tundra under global warming

**DOI:** 10.1111/gcb.14935

**Published:** 2020-01-20

**Authors:** Andrea Ghirardo, Frida Lindstein, Kerstin Koch, Franz Buegger, Michael Schloter, Andreas Albert, Anders Michelsen, J. Barbro Winkler, Jörg‐Peter Schnitzler, Riikka Rinnan

**Affiliations:** ^1^ Research Unit Environmental Simulation (EUS) Institute of Biochemical Plant Pathology Helmholtz Zentrum München Neuherberg Germany; ^2^ Terrestrial Ecology Section Department of Biology University of Copenhagen Copenhagen Denmark; ^3^ Institute of Biochemical Plant Pathology (BIOP) Helmholtz Zentrum München Neuherberg Germany; ^4^ Research Unit for Comparative Microbiome Analysis (COMI) Helmholtz Zentrum München Neuherberg Germany; ^5^ Center for Permafrost Department of Geoscience and Natural Resource Management University of Copenhagen Copenhagen Denmark

**Keywords:** ^13^CO_2_, Arctic, climate change, de novo biosynthesis, global warming, net ecosystem exchange, subarctic heath, terpene, tundra, volatile organic compound

## Abstract

Warming occurs in the Arctic twice as fast as the global average, which in turn leads to a large enhancement in terpenoid emissions from vegetation. Volatile terpenoids are the main class of biogenic volatile organic compounds (VOCs) that play crucial roles in atmospheric chemistry and climate. However, the biochemical mechanisms behind the temperature‐dependent increase in VOC emissions from subarctic ecosystems are largely unexplored. Using ^13^CO_2_‐labeling, we studied the origin of VOCs and the carbon (C) allocation under global warming in the soil–plant–atmosphere system of contrasting subarctic heath tundra vegetation communities characterized by dwarf shrubs of the genera *Salix* or *Betula*. The projected temperature rise of the subarctic summer by 5°C was realistically simulated in sophisticated climate chambers. VOC emissions strongly depended on the plant species composition of the heath tundra. Warming caused increased VOC emissions and significant changes in the pattern of volatiles toward more reactive hydrocarbons. The ^13^C was incorporated to varying degrees in different monoterpene and sesquiterpene isomers. We found that de novo monoterpene biosynthesis contributed to 40%–44% (*Salix*) and 60%–68% (*Betula*) of total monoterpene emissions under the current climate, and that warming increased the contribution to 50%–58% (*Salix*) and 87%–95% (*Betula*). Analyses of above‐ and belowground ^12/13^C showed shifts of C allocation in the plant–soil systems and negative effects of warming on C sequestration by lowering net ecosystem exchange of CO_2_ and increasing C loss as VOCs. This comprehensive analysis provides the scientific basis for mechanistically understanding the processes controlling terpenoid emissions, required for modeling VOC emissions from terrestrial ecosystems and predicting the future chemistry of the arctic atmosphere. By changing the chemical composition and loads of VOCs into the atmosphere, the current data indicate that global warming in the Arctic may have implications for regional and global climate and for the delicate tundra ecosystems.

## Graphical abstract

We studied the origin of biogenic volatile organic compounds (VOCs) and carbon (C) allocation under global warming in subarctic heath tundra ecosystem using isotope labeling of ^13^CO_2_. Our results show the importance of de novo monoterpene biosynthesis and the impact of warming in vegetation communities characterized by *Salix* spp. (willows) or *Betula* spp. (birch). Warming increased overall VOC emissions and altered the composition of the volatile blend toward more reactive compounds. Analyses of above‐ and belowground ^12/13^C suggest shifts of C allocation and negative effects of warming on C sequestration in these delicate tundra ecosystems.

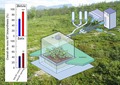

## 1 INTRODUCTION

In the Arctic, a temperature increase of 1°C per decade has been measured for the last 30 years (IPCC, 2013), which is twice the increment relative to the global average. Current models estimate the arctic surface temperature has increased 3–11°C compared to the preindustrial age. In the subarctic regions, global warming already impacts plant productivity and biomass allocation, plant species distribution (Elmendorf et al., 2012), soil properties (Rinnan, Michelsen, Bååth, & Jonasson, 2007), precipitation patterns (Callaghan et al., 2011), and emissions of biogenic volatile organic compounds (VOCs; Rinnan, Steinke, McGenity, & Loreto, 2014). On the global scale, VOCs and in particular, volatile terpenoids, are reactive compounds that play crucial roles in atmospheric processes (Claeys et al., 2004; Ehn et al., 2014; Fuentes et al., 2000; Ghirardo et al., 2016; Goldstein, Koven, Heald, & Fung, 2009; Guenther, 2013; Pun, Wu, & Seigneur, 2002). Due to the size of subarctic regions and the much stronger impact of global warming on VOC emissions there compared to lower latitudes (Kramshøj et al., 2016; Lindwall, Schollert, Michelsen, Blok, & Rinnan, 2016; Lindwall, Svendsen, Nielsen, Michelsen, & Rinnan, 2016; Schollert, Burchard, Faubert, Michelsen, & Rinnan, 2014), changes in subarctic VOC emissions may affect climate on regional and global scales. The biochemical mechanisms underlying the temperature‐dependent VOC emission from subarctic regions are, however, still largely unknown (Tang et al., 2016).

Emissions of plant terpenoids can occur immediately after their biosynthesis in the mesophyll (de novo emission) of foliage, or it originates from the evaporation of compounds from inner (e.g., resin ducts) or outer (e.g., glandular trichomes) specialized storage tissues (pool emission; Loreto & Schnitzler, 2010). De novo emissions of terpenoids rely on photoassimilates and follow light‐ and temperature‐dependent processes of photosynthesis (Ghirardo et al., 2010). Pool emissions are largely controlled by temperature, whereby liquid terpenoids evaporate. To model terpenoid emissions from the Subarctic during the rapidly proceeding warming, it is paramount to study the processes controlling the emissions and quantify to what extent future increasing temperatures enhance de novo biosynthesis and the evaporation rate of volatile emissions from storage pools. The use of ^13^C stable isotope techniques and laboratory studies under controlled conditions, allows for comprehensive investigations of terpenoid carbon sources to link biosynthesis and emission (Ghirardo, Gutknecht, Zimmer, Brüggemann, & Schnitzler, 2011; Ghirardo et al., 2010, 2014). Field studies alone, in the form of correlation analysis of VOC emissions and temperature, are not sufficient to decipher the de novo synthesis from the pool emissions (Ghirardo et al., 2010; Taipale et al., 2011; Wu et al., 2017). Understanding the processes behind terpenoid emissions is essential to mechanistically connect environmental factors to VOC emissions when building mathematical models for the prediction of future VOC budgets for terrestrial ecosystems (Arneth & Niinemets, 2010; Grote et al., 2006; Grote & Niinemets, 2008; Guenther, 2013; Harrison et al., 2013; Monson, Grote, Niinemets, & Schnitzler, 2012).

In the present study, we elucidate the origin of plant volatile emissions from high latitude tundra ecosystems. We studied two contrasting vegetation communities, characterized by either *Salix myrsinites* or *Betula nana* as the dominant deciduous plant species. Mesocosms, that is, blocks of tundra soil and the intact vegetation on top, were collected from the Subarctic and grown under highly controlled environmental conditions in climate chambers of a phytotron facility, which allows the realistic simulation of climate and solar radiations of UV‐Vis‐NIR (Döhring, Köfferlein, Thiel, & Seidlitz, 1996; Seckmeyer & Payer, 1993; Thiel et al., 1996; Vanzo et al., 2015). Using ^13^CO_2_‐labeling technique and this chamber system, we deciphered and quantified the “de novo” and the “pool” parts from the total terpenoid emissions (Ghirardo et al., 2010; Harley, Eller, Guenther, & Monson, 2014) under simulated actual and predicted future (IPCC, 2013) summer temperatures of the subarctic regions. Comparison of the VOC emissions from the tundra mesocosms under the two climate scenarios show an impact of global warming on VOC patterns and emission potentials. Furthermore, by tracing the airborne ^13^C within the mesocosms, this study reveals the differential carbon allocation patterns of plant species above‐ and belowground.

## 2 MATERIALS AND METHODS

### 2.1 Plant material and sampling of mesocosms

In July 2014, 48 mesocosms were collected from a mesic subarctic heath in Abisko (68.3495°N, 18.8304°E), Sweden. The mesocosms were representative of the natural heath tundra and are heterogenic; some with very dense and others with less vegetation cover. The collection site was close to an experimental field site where soil characteristics (Lett & Michelsen, 2014; Rinnan, Michelsen, & Jonasson, 2008) and emissions of VOCs (Tiiva et al., 2008; Valolahti, Kivimäenpää, Faubert, Michelsen, & Rinnan, 2015) have been previously reported. The site was within 2 km of the Abisko Scientific Research Station, where climate data are collected (https://polar.se/en/research-in-abisko/research-data/). Mesocosm collection was performed by cutting and digging out an 18 × 18 × 10 cm (W × D × H) piece of soil containing plants and accommodated into quadratic polyethylene terephthalate pots of the same size. We collected two types of mesocosms, containing several plant species and differing in the quantity of *B. nana* L. (abbr. “B”) or *S. myrsinites* L. and *Salix reticulata* L. (abbr. “S”; see Figure S1). The major plant species common to both mesocosm types were *Empetrum nigrum* ssp. *hermaphroditum* (Hagerup) Böcher, *Andromeda polifolia* L., *Vaccinium* spp., and *Carex* spp. (see Table S1 for details). The soil is highly organic and has a pH of ~7 (Rinnan et al., 2008). Mesocosms were transported within 2 weeks of collection to the phytotron chambers of the Helmholtz Center in Munich, Germany.

### 2.2 Experimental setup and climate simulation

The 48 mesocosms were randomly split into two groups and placed in two respective phytotron chambers for the simulation of the actual and future climates. The walk‐in phytotron is composed of unique climate chambers that allow a realistic reproduction of climate, including the simulation of solar radiation spectra of UV‐Vis‐NIR (Döhring et al., 1996; Seckmeyer & Payer, 1993; Thiel et al., 1996). Overall, the subchambers are adequate for gas‐exposure experiments (Kozovits, Matyssek, Blaschke, Göttlein, & Grams, 2005), and analyses of gas‐exchange of CO_2_, H_2_O, and VOC emissions, as previously described in detail (Vanzo et al., 2015). Each of the two chambers contains four Plexiglas subchambers (size: 0.8 × 1.0 × 1.1 m; W × D × H; Luedemann, Matyssek, Winkler, & Grams, 2009), hosting six mesocosms each. The subchambers were continuously flushed by ~670 L/min of purified air. The phytotron air is cleaned by molecular filtration, ozone treatments, and chemisorption: air is filtered from PM1 and PM10 using M5 and F9 molecular filters (EN 779:2012; Camfil KG), mixed with ~1 ppmv O_3_ and passed through four blocks of filters (1 m^3^ each), containing porous pellets (3 mm) of activated carbon (A) and activated alumina granules impregnated with potassium permanganate (KMNO_4_; P; HS‐Activated Carbons & HS‐Clean Pro, both from HS‐Luftfilterbau GmbH). Finally, air is filtrated by H13 filter (EN 1822:2009). The sequence of filtration is: M5‐F9‐A‐P‐P‐A‐H13. At the inlet, the resulting zero‐levels of NOx and O_3_ were continuously monitored (AC31M and O341M, ENVEA).

**Figure 1:**
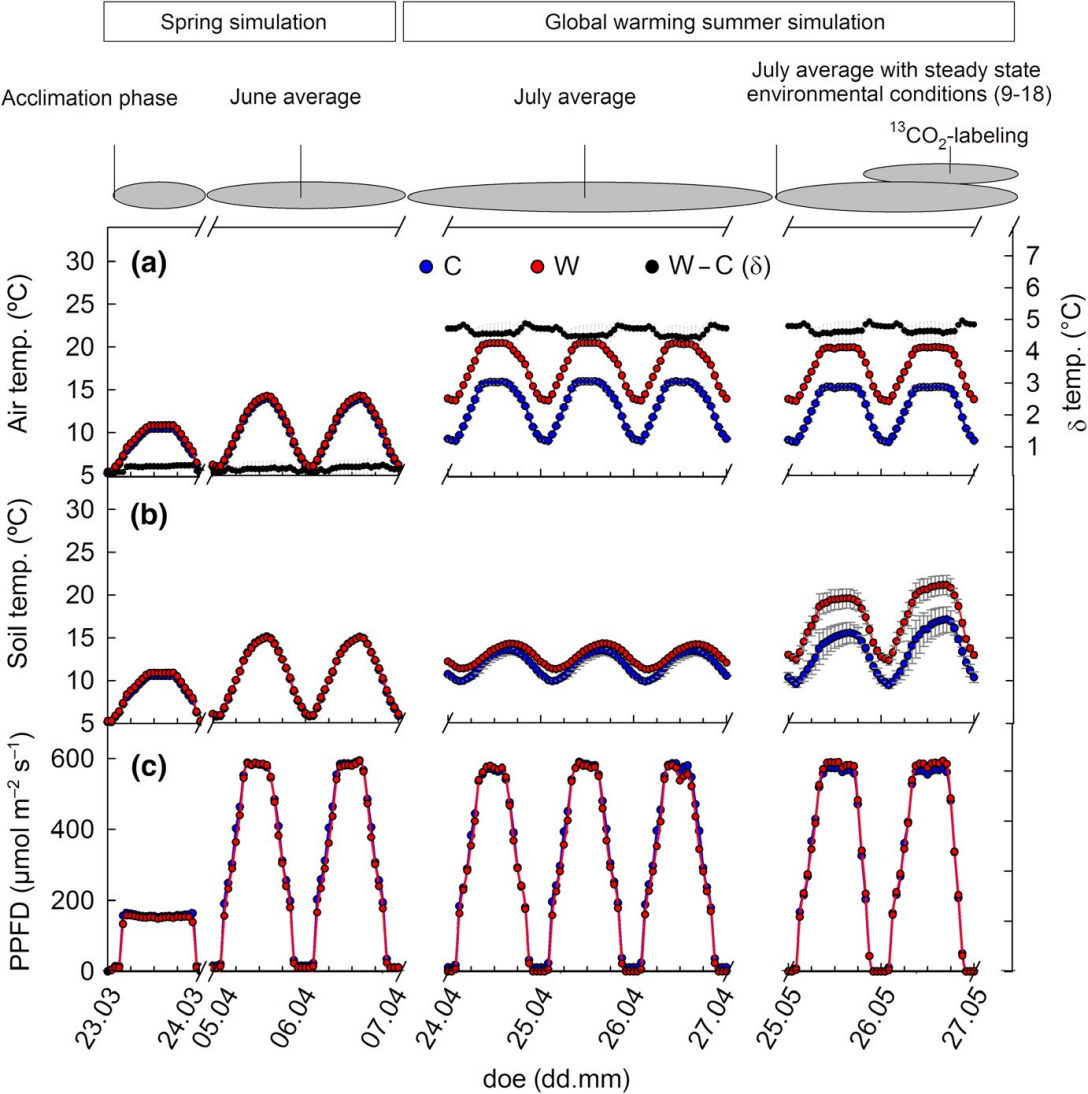
Experimental design and environmental conditions of (a) air temperature, (b) soil temperature at 0–2 cm depth, and (c) light intensities (photosynthetically active quantum flux density [PPFD]) at canopy level. The simulation of spring climate for the gradual adaptation of the mesocosms was followed by the simulation of actual summer climate (C, in blue) and future predicted warming climate scenario (W, in red). ^13^CO_2_ labeling experiments were performed during the last 2 weeks under steady state environmental conditions of light and temperature between 9 and 18 (CET). Data depict representative days for each climate phase. Mean values of four chambers ± *SE*. Differences between warming and control temperatures (‘W − C’) are referred to as delta (δ, in black). C, control; doe, day of experiment; W, warming

To recover from transport and potential mechanical stress during sampling, the mesocosms were cultivated in the phytotron under conditions representative for autumn in Abisko until late October. Plant dormancy occurring in arctic winter under snow was achieved by moving the mesocosms at the end of October to a dark room where they were maintained at 3–4°C until the end of March. Irrigation was regularly performed during the recovery period to maintain soil moisture at approx. 50%. Mesocosms were returned to the phytotron at the end of March and dormancy was gradually released by simulating subarctic spring conditions, which started on March 23 and included a 5‐day long initial phase (“acclimation phase”, Figure 1), where maximum daytime irradiation intensities were increased gradually (see Figure 1 for environmental parameters). For the spring season, the June climatic conditions in Abisko were used, based on 10 year hourly average climate (maximum incident photosynthetically active quantum flux density (PPFD) levels of 922 µmol m^−2^ s^−1^, min/max air temperature of 5.7/11°C). These conditions were applied for 10 days and were followed by initiation of the summer season concomitant with the warming treatment. We simulated the summer climate in the control (C) chamber by using the most recent 10 year average climate data of July in Abisko and increased the air temperatures by 5°C in the second chamber to simulate the predicted warming scenario (W). To resemble the warming of the subarctic permafrost soil region, soil was cooled from belowground to the same temperature (4°C) while soil surface temperatures increased due to air temperatures. The resulting temperature differences between W and C top soils at 0–2 cm depth were 2.72 ± 0.32°C (mean ± *SD*; night, 23–02, times are always referred to in CET) and 3.22 ± 0.68°C (day, 13–16). Soil temperatures at 2–5 cm depth were 1.80 ± 0.3 (night) and 0.69 ± 0.16°C (day), during 2 weeks before the ^13^C‐labeling (Figure S2d). Irradiation was similar for C and W throughout the whole experiment with a PPFD at canopy height of 600 µmol m^−2^ s^−1^ (Figure 1c). The July simulation was applied for approx. 1 month.

Overall, the experimental procedure ensured that mesocosms gradually entered the spring and summer season, as indicated by greening of foliage and the development of flowers. We observed that mosses did not recover well, likely due to the lack of precipitation inside the chambers. Therefore, mosses were assumed not to be fully active during the experiments.

### 2.3 ^13^C‐labeling of individual mesocosms

One week prior to labeling the mesocosms with ^13^CO_2_, we changed the environmental conditions to constant PPFD and air temperature between 9 and 18 CET, which were maintained during the labeling experiment. The purpose was to reach a metabolic steady‐state, as a precondition for studying the incorporation of ^13^C into terpenoids (Ghirardo et al., 2014). The air temperature difference between C and W chambers remained at the targeted temperature of 5°C (4.8 ± 0.3°C actual difference), while soil temperature difference increased to 2.6 ± 1.6°C during the day, due to the prolonged maxima of light and temperature.

The ^13^CO_2_‐labeling of individual mesocosms was performed using a six‐cuvette system installed inside the phytotron to improve VOC detection (Figure S3). The ^13^C‐labeling procedure followed established protocols (Ghirardo et al., 2010, 2011, 2014). Briefly, each cuvette was continuously flushed with VOC‐free synthetic air and mixed with 99% of either ^12^CO_2_ (prelabeling and control) or ^13^CO_2_ (during labeling). The airflow was 650 ml/min and the final CO_2_ concentration at the inlet was set to 450 ppmv, to maintain a CO_2_ concentration in the cuvette of ~350 ppmv (min. 320 ppmv) during VOC sampling (see also Figure S3 for more details). This procedure was necessary to avoid CO_2_ depletion inside the cuvette under low airflow, which may cause erroneous terpenoid measurements due to the negative correlation between CO_2_ concentrations and terpenoid emissions (Rosenstiel, Potosnak, Griffin, Fall, & Monson, 2003; Way et al., 2013; Wilkinson et al., 2009).

To avoid mechanical disturbance of the plant terpenoid pool emissions during labeling (Ghirardo et al., 2010), 36 mesocosms were individually enclosed within cuvettes between 17:30 and 18:30, the day before the labeling took place. On the day of labeling, ^13^CO_2_ was supplied between 11:00 and 16:30. Finally, ^12^CO_2_ was used for an additional hour (until 17:30) before stopping all measurements and placing the mesocosms outside the cuvettes in the phytotron subchambers. The residence time of ^13^CO_2_ in the system was ~4.5 min, and the complete washout (>99%) of CO_2_ from the cuvette was observed 46 min after changing the CO_2_ source. This protocol ensured the complete measurements of ^13^C into the de novo synthetized VOCs and minimized the variability of ^13^C due to different sampling times. As a control for the labeling experiment, the same procedure was performed but using ^12^CO_2_ exclusively as a source of CO_2_. Background measurements were conducted before and after the experiment using cuvettes containing empty pots. The signals obtained from the background measurements were subtracted from those of samples.

Mesocosms were harvested at 11:00 the day after the labeling by cutting the aboveground part of the plants. Plant material was sorted for species, plant tissues were divided into leaves and stems (where possible), and all samples were dried at 68°C for 48 hr for the determination of dry biomass (see Table S1). Roots were sorted from soil by hand and divided into fine (<2 mm) and coarse fractions. Soil samples contained a mix of three soil layers (1:1:1, weight), containing the top 1 cm, mid, and bottom layers. Samples were finely ground and divided into fractions for further analyses.

### 2.4 Online VOC measurements using PTR‐MS

Volatile organic compound emissions were measured both from subchambers and cuvettes. Chamber‐enclosed multiple mesocosms were monitored before the ^13^C‐labeling experiment and during the preadaptation phase of mesocosms using the high‐sensitivity proton‐transfer‐reaction quadrupole mass spectrometer (PTR‐QMS; Ionicon Analytik GmbH). The instrument was operated as previously described in detail (Ghirardo et al., 2010, 2011; Kreuzwieser et al., 2014) in combination with the phytotron (Vanzo et al., 2015).

Online measurements of VOCs of individual, cuvette‐enclosed mesocosms were achieved during the ^13^C‐labeling experiments using a proton‐transfer‐reaction time‐of‐flight mass spectrometer (PTR‐ToF‐MS, Ionicon; Jordan et al., 2009) by drawing an aliquot of air sample (~120 ml/min). The instrument was operated with an *E*/*N* of 130 Td (*E* = the electric field strength, *N* = the gas number density; 1 Td = 10^−17^ Vcm^2^; drift tube (dt) pressure = 2.2 mbar; dt voltage = 500 V, dt temperature = 90°C). Throughout the experiments, the ions H_3_O H_2_O^+^, O_2_^+^, and NO^+^ were kept below 10%, 3%, and 0.2% of the primary ions, respectively. Calibration of the instrument was achieved by humidity‐dependent dilution (0%–90% RH at 24°C) procedures performed with 12 different concentrations ranging between 0 and 150 ppbv of an 11 VOC standard mixture (Apel‐Riemer Environmental), passed through the entire system as described before (Ghirardo et al., 2011). Sesquiterpenes and isotopologue compounds of ^13^C were quantified using empirical sensitivities based on relative transmission factor (Taipale et al., 2008) calculated from instrumental sensitivities and obtained from measuring ions originating from a 17 VOC standard mixture (Ionicon). This calibration procedure agreed with data obtained from the GC‐MS analysis. The uncertainty given by the gas standard and the calibration procedures was calculated to be <10%. The relative mass accuracy and precision (σ) at *m*/*z* 137.133 (monoterpenes) were 0.6 and 2.6 ppm at concentration of 10 ppbv, respectively. Limits of detection (LOD) were calculated with 2σ and ranged between 0.16 ppbv (isoprene) and 6.01 ppbv (ethanol; Table S2). The accuracy of the measurements was ± 6%. The response time throughout the whole system was less than 20 s.

Each cuvette was measured for 5 min before switching to the next cuvette. The first 3 min of measurements was used as flushing time and the corresponding MS acquisition data were disregarded from the data analysis, to remove any interference from the previous cuvette sampling. The remaining 2 min containing six measurement points (20 s integration time per PTR‐ToF‐MS cycle) were averaged and used for the calculation of VOC fluxes as previously described (Ghirardo et al., 2011), based either on total dried vascular plant biomass (foliage plus stems, g^−1^) or on ground area (m^2^). Therefore, the entire measurement cycle through all six cuvettes took 30 min. Data are presented at 1 hr time resolution.

### 2.5 Offline VOC analysis using GC‐MS

Volatile organic compounds emitted from individually cuvette‐enclosed mesocosms were collected for GC‐MS analysis by passing air (100 ml/min for 60 min) through glass cartridges filled with 40 mg Tenax TA 60/80 and 40 mg Carbopack X 40/60 (both from Sigma‐Aldrich; see also Figure S3). Samples were collected immediately before labeling (09:45–10:45), and during the last hour of the ^13^C‐labeling (i.e., 15:30–16:30; Figure 3). Quantitative and qualitative VOC analysis was achieved as previously described (Ghirardo, Heller, Fladung, Schnitzler, & Schroeder, 2012; Ghirardo et al., 2016; Weikl, Ghirardo, Schnitzler, & Pritsch, 2016). The procedure was optimized by changing the following parameters: Samples were thermally desorbed by increasing the temperature from 35 to 270°C at a rate of 280°C/min, then cryo‐refocused on Tenax TA at −50°C for 0.31 min, and reinjected by ramping the temperature to 270°C at a rate of 12°C/s and holding for 2 min. The temperature of the GC oven started at 40°C, increased to 80°C at a rate of 6°C/min and held for 3 min, then increased to 170°C at 3.4°C/min, and from 170°C to 300°C at 12°C/min and held for 4 min. Each sample contained 859.3 pmol of δ‐2‐carene as the internal standard and the GC‐MS cartridge was dried with ultrapure helium before analysis.

The chemical identification of the VOCs was based on samples collected under ^12^CO_2_. The peaks found in labeled samples at the same retention time and having a consistent fragmentation pattern enriched in the isotopologue fragments were assumed to be the same chemical compounds as those in unlabeled samples. The quantification of VOCs was achieved by performing a calibration curve using six different concentrations of pure standard mixtures, independently created in triplicate. The standard mixtures contained the monoterpenes α‐pinene, myrcene and limonene, oxygenated monoterpenoids linalool and eucalyptol (both classes referred as MT), sesquiterpenes β‐caryophyllene and *E*‐(β)‐farnesene, as well as oxygenated sesquiterpenoids nerolidol and farnesol (both referred as sesquiterpenes [SQT]). The recorded MS signals were linear (*r* ^2^ = .986–.9993) for the range of 0–900 pmol, which covered the sampled air concentrations. Volatiles that were not available as standards were quantified using calculated response factors (Kreuzwieser et al., 2014). The calculated response factors of available standards (alkane, terpene, benzenoids) had an uncertainty typically of 1%–3%, occasionally <8%. No significant differences were found among blank tubes, therefore the mean of all background measurements was used for the final background correction. The LOD were set to 2*σ and limits of quantification to five times their respective LOD.

### 2.6 Calculation of ^13^C‐incorporation into VOCs from GC‐MS spectra

The mass spectra recorded for each single peak were extracted using the Enhanced ChemStation software (Version G1701EA, Revision E.02.01; Agilent Technologies) and further analyzed using Excel. Prior to export, averages of ~6–20 mass scans were computed around maximum peak height, and background corrected using the average of a similar number of scans immediately before the beginning of the peak. The abundances of each *m*/*z* ion were rounded at a mass resolution of 0.1 amu. The sum of the abundances covering the *m*/*z* range of the isotopologue masses of each parent ion (i.e., 68–73 for isoprene, 136–146 for MT, 154–164 for oxygenated MT, 204–219 for SQT) was used to calculate the percentage of the ^13^C‐incorporation into the VOC, or atomic percentage excess above the natural abundance of ^13^C, as follows:

(1) $$\text{At}\%{}^{13}C\;=\;\left[\frac{\sum_{i=1}^{n}\left(A_{i}\right)\cdot \;i\cdot 100}{\sum_{i=0}^{n}\left(A_{i}\right)\cdot \;n}\right]-1.1,$$

where *A_i_* is the abundance of the isotopologue mass containing *i* ^13^C and *i* = 0 refers to the parent mass having only ^12^C, *n* is the number of the C atom in the skeleton of the VOC, and 1.1 (in percent) is the rounded average of the natural abundance of ^13^C commonly found in biological samples.

The probability *p* of the naturally appearing ^13^C isotopologues was calculated according to the probability mass function (e.g., Karl et al., 2002) as follows:

(2) $$p\left(k\right)=\left(\begin{array}{c} n\\ k \end{array}\right)\cdot p^{k}{\left(1-p\right)}^{n-k},$$

where *n* and *k* are the numbers of ^12+13^C and ^13^C atoms inside the compounds, respectively.

### 2.7 Calculation of atmospheric C‐incorporation into VOC and de novo biosynthesis of monoterpenes

To link recently fixed carbon from photosynthesis to VOC biosynthesis, the ^13^CO_2_‐labeling approach was employed (Ghirardo et al., 2011, 2014; Loreto, Ciccioli, Brancaleoni, Frattoni, & Delfine, 2000) and the de novo biosynthesis of monoterpenes was calculated as previously described (Ghirardo et al., 2010). Because microbial and root respiration processes occurring belowground were significant sources of ^12^CO_2_, which in turn diluted the ^13^CO_2_ concentrations during the labeling experiment, the true percentage of ^13^CO_2_ inside the cuvette was calculated as follows:

(3) $${}^{13}{\text{CO}}_{2}\left[\%\right]=100-\left[\frac{(\delta {\text{CO}}_{2}-\text{ZP})\times 100}{{\text{CO}}_{2i}+\delta {\text{CO}}_{2}}\right],$$

where δCO_2_ is the CO_2_ contribution of soil (microbial and root) respiration, ${\text{CO}}_{2i}$ is the concentration at the inlet, and ZP is the zero point of the cuvette (equal to δCO_2_ recorded on empty cuvette). We estimated δCO_2_ by night measurements (22–23, PPFD = ~50 µmol m^−2^ s^−1^), that is, when net ecosystem CO_2_ exchange was negative (see Figure 3). The resulting percentages of calculated ^13^CO_2_ inside the cuvette were 63.1 ± 0.9 (“*Betula*”, C), 62.3 ± 2.5 (“*Betula*”, W), 66.7 ± 2.2 (“*Salix*”, C), and 60.9 ± 2.6 (“*Salix*”, W) and agreed with ^12^CO_2_ signals measured with the infrared gas analyzer (IRGA). For validation, we applied the values obtained to calculate the incorporation of atmospheric CO_2_ into isoprene emitted under control conditions from mesocosms characterized by *Salix* spp., which are strong isoprene emitters. The obtained values indicated that mesocosms dominated by *Salix* spp. use 79.9 ± 0.9% of atmospheric CO_2_ for the biosynthesis of isoprene, which agrees well with literature (Ghirardo et al., 2010, 2011, 2014; Karl et al., 2002; Schnitzler et al., 2004).

### 2.8 Net ecosystem CO_2_ exchange

Gas‐exchange of CO_2_ was measured by IRGA (GFS‐3000; Heinz Walz GmbH) and mesocosm net ecosystem exchange (NEE) was calculated according to the equation of von Caemmerer and Farquhar (1981). NEE, as a measure of flux from the atmosphere to the ecosystem, has positive values for net ecosystem uptake. During ^13^CO_2_‐labeling, when IRGA is sensitive to ^13^C isotope, the NEE was estimated based on measurements performed on ^12^CO_2_ (control runs). The percentage changes of NEE from the hourly mean at 8:00 (i.e., approx. at NEE max) were calculated from ^12^CO_2_ control runs and the resulting values were applied to the labeled samples during ^13^CO_2_ exposure. The values were calculated individually for each mesocosm type and climate condition.

### 2.9 ^12/13^C isotopic composition within the mesocosm, soil and microbial analyses, and C‐allocation study

Soil samples were aliquoted for dry soil analyses and extraction. For the ^12/13^C isotopic pattern and C and N concentrations of soil and plant material, approx. 5 mg of finely ground sample was packed in tin capsules and analyzed on an isotope ratio mass spectrometer (IRMS; Isoprime Ltd) coupled to a Eurovector CN elemental analyzer (Ravn, Ambus, & Michelsen, 2017; Ravn, Elberling, & Michelsen, 2017).

Microbial biomass C was determined from fresh soil material by the chloroform fumigation extraction method using 0.1 g dry soil each and 10 ml 0.01 M CaCl_2_ solution. Nonfumigated controls were used to assess the dissolved organic carbon content in soil (DOC). The measurements were carried out with a DOC/TNb‐Analyzer (Dimatoc 2000; Dimatec Analysen GmbH). The differences between fumigated and nonfumigated extracts provide the concentrations of microbial biomass, using the extraction yields of 0.45 (Joergensen, 1995). δ^13^C in the DOC‐extracts were measured by LC‐IRMS with an MAT 253 coupled to a LC IsoLink‐Interface (Thermo Fisher) described by Krummen et al. (2004). δ^13^C of microbial biomass was calculated as follows:

(4) $$\delta {}^{13}\text{C}=\frac{\left({\text{C}}_{\text{fum}}\times \delta {}^{13}{\text{C}}_{\text{fum}}\right)-\left({\text{C}}_{\text{nfum}}\times \delta {}^{13}{\text{C}}_{\text{nfum}}\right)}{\left({\text{C}}_{\text{fum}}-{\text{C}}_{\text{nfum}}^{}\right)},$$

where C_fum_ and C_nfum_ are the concentrations (mg/g) of C in the fumigated and nonfumigated soils, respectively (Marx, Buegger, Gattinger, Zsolnay, & Munch, 2007).

To assess the C‐allocation within the mesocosms from recently fixed atmospheric CO_2_, the sum of fixed ^13^CO_2_ during labeling was calculated using the estimated NEE and the percentage of ^13^CO_2_ inside the cuvette (previous section).

The ^13^C‐allocation into VOC (*A*_VOC_) was calculated as the total integrated emission of ^13^C‐VOC between starting (s) and after (a) 1 hr of ^13^CO_2_‐labeling:

(5) $$A_{\text{VOC}}=\int_{s}^{a}\Phi \left(t\right)dt,$$

where Φ(*t*) is the VOC emission rate at time *t*. To calculate the numerical integration, the trapezoidal rule was used. The residence time and the washing out of the CO_2_ throughout the whole system was taken into account. Adding one extra hour at the end of ^13^CO_2_‐labeling procedure allowed for complete measurement (>99%) of the C‐allocation into the de novo VOC biosynthesis.

### 2.10 Statistical analysis

The four subchambers per climate, containing the two mesocosm types S and B, served as the units of replication (*n* = 4). For the ^13^C‐labeling experiment, six cuvette‐enclosed mesocosms per temperature treatment and vegetation type were labeled (*n* = 6), and three mesocosms per temperature and vegetation type were used as labeling controls (*n* = 3). NEE and VOC emission rates data were composed of both labeling and control experiments (*n* = 9). In total, 36 mesocosms were used in the ^13^C‐labeling experiment. *T* test, one‐way, and two‐way ANOVA were performed using the software package Sigma‐plot (v11.0; Systat Software Inc.). Statistically significant differences were tested at *p* < .05, but tendencies toward significance (.05 < *p* < .1) are also reported. Multivariate data analysis was performed following established procedures (Ghirardo, Sørensen, Petersen, Jacobsen, & Søndergaard, 2005; Ghirardo et al., 2016; Jud et al., 2016; Weikl et al., 2016) using the software package SIMCA‐P version 13.0 (Umetrics). Orthogonal partial least square regression (OPLS) was implemented as described elsewhere (Riedlmeier et al., 2017).

## 3 RESULTS

### 3.1 Effects of warming on VOC emissions

#### 3.1.1 Chamber measurements

**Figure 2:**
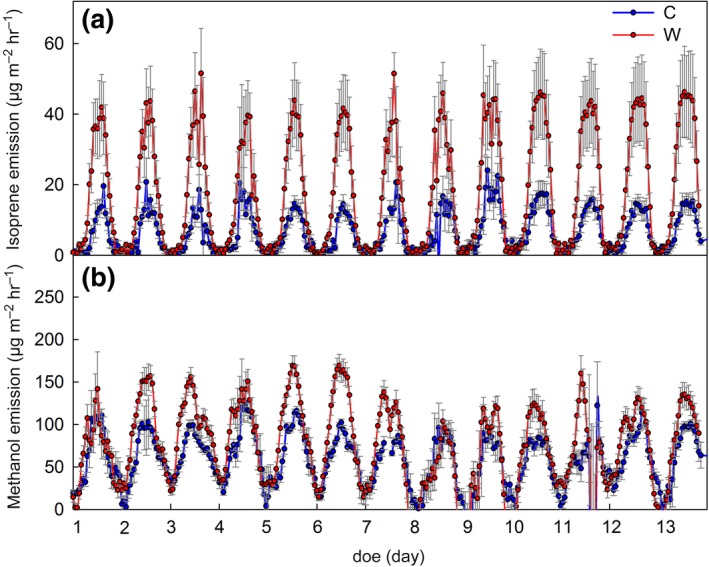
(a) Isoprene and (b) methanol emission rates from mixed *Betula* and *Salix* mesocosms growing under control (C, in blue) and warming (W, in red) climate. Data depict the emissions from 48 mesocosms enclosed in eight subchambers (i.e., six mesocosms per subchamber) of the phytotron for 13 days before starting the ^13^C‐labeling, and measured by PTR‐QMS. Data are normalized for mesocosm ground area to realistically simulate the subarctic ecosystem emissions. Means of *n* = 4 (subchambers) ± *SE*. doe, day of experiment

To study the VOC emissions from subarctic heath tundra under future predicted global warming conditions, we simulated the mean summer season of Abisko, Sweden in July, and we increased the air temperature by +5°C compared to the control (Figure 1a, mid panel). For both *Betula* (B) and *Salix* (S) mesocosms, the warming treatment significantly increased isoprene (*p* < .001, ANOVA) and methanol emissions (*p* = .003; Figure 2). During 2 weeks prior to the ^13^C‐labeling experiment, isoprene and methanol emission rates increased by 2.8 and 1.4 times under warming relative to the control, respectively. More carbon (C) was emitted as methanol than as isoprene, and the methanol‐to‐isoprene C ratio changed from 3.0 under control to 1.4 under warming. The detection of volatiles other than isoprene and methanol was poor, due to the dilution from the high inlet airflow (~670 L/min) in the subchambers. Nevertheless, the increase in emissions, together with the decrease (*p* < .001) in the methanol‐to‐isoprene carbon atom ratios (Figure S4), indicate that the chemical atmospheric composition in the Subarctic might change and that the C‐loss from heath tundra as VOC will increase in future.

#### 3.1.2 Cuvette measurements

**Figure 3:**
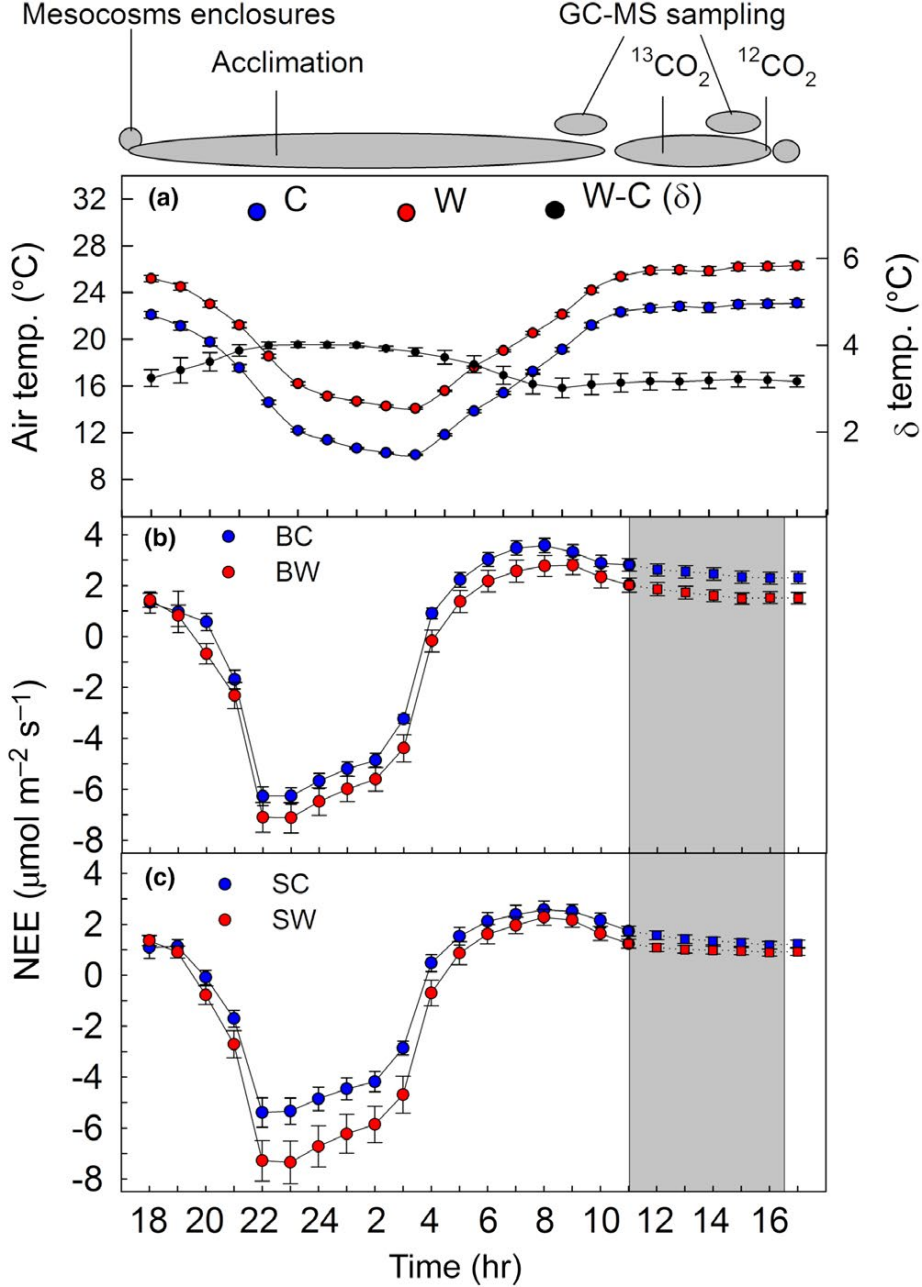
(a) Hourly timeline of the ^13^CO_2_‐labeling experiment and cuvette air temperatures. (b, c) Net ecosystem CO_2_ exchange (NEE) of the two mesocosm types, “*Betula*” (panel b) and “*Salix”* (panel c) under control (in blue) and warming (in red) climate conditions. Measurements were performed on the individual, cuvette‐enclosed mesocosms. For the ^13^CO_2_‐labeling phase (in gray), NEE is estimated (dash lines). (a) Temperature differences between W and C (in panel a) are referred as delta (δ, in black) and were statistically significant at *p* < .001. Significant main effects of warming climate and mesocosm type were tested with two‐way ANOVA (*p* < .05). Data depict mean values of (a) *n* = 18, and (b, c) *n* = 9 ± *SE*. B, *Betula*; C, control; S, *Salix*; W, warming. Positive values of NEE represent net ecosystem uptake of CO_2_ from the atmosphere to the ecosystem

**Figure 4:**
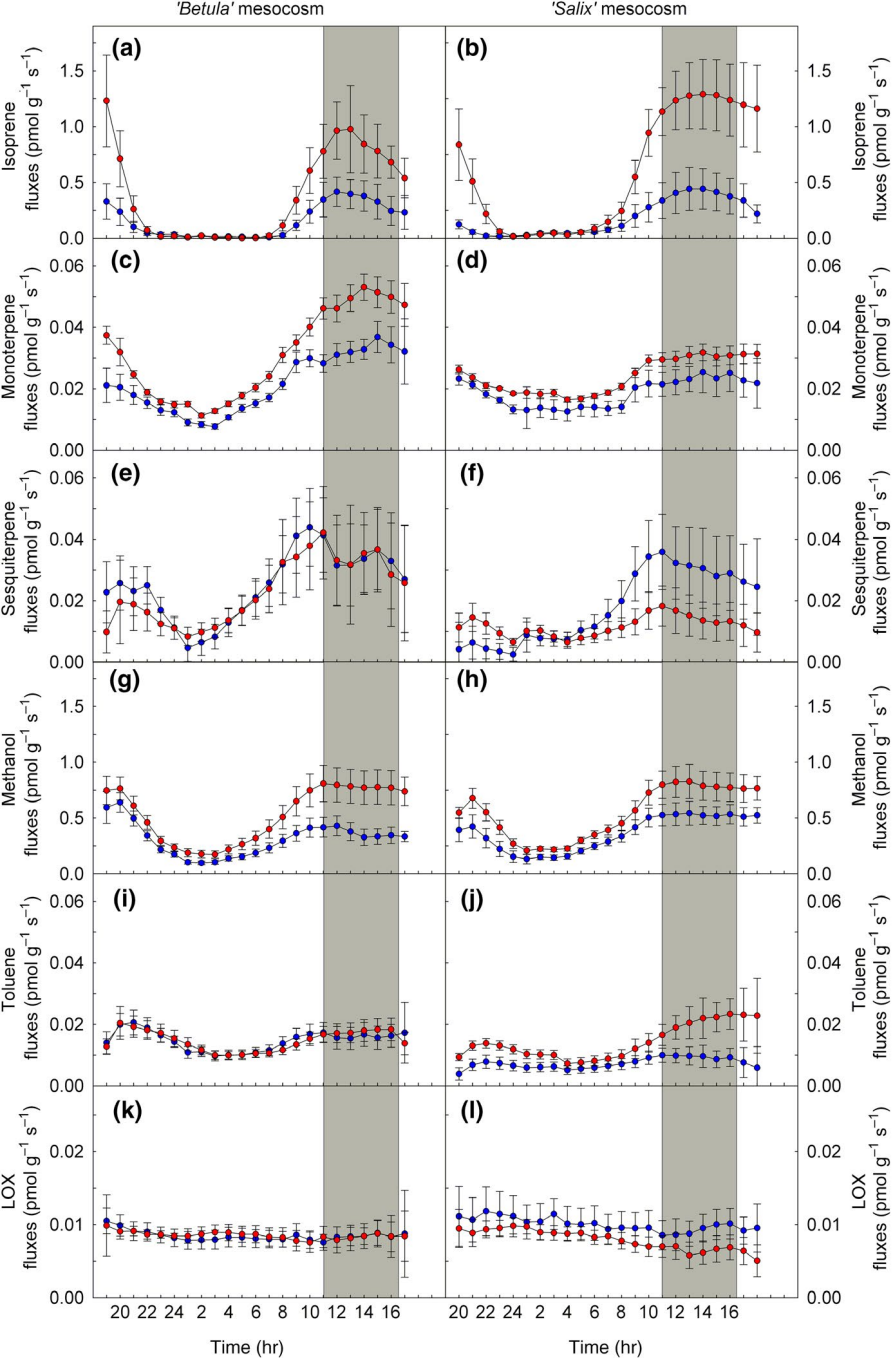
Time‐course emissions of (a, b) isoprene, (c, d) monoterpenes, (e, f) sesquiterpenes, (g, h) methanol, (i, j) toluene, and (k, l) lipoxygenase (LOX) products from “*Betula*” (left panels) and “*Salix*” (right panels) mesocosms under control (in blue) and warming (in red) climate conditions. Measurements were performed using PTR‐ToF‐MS on the individual, cuvette‐enclosed mesocosms. For each volatile organic compound, data depict the sum of all the corresponding isotopologues measured masses. The ^13^CO_2_‐labeling phase is shown in gray. Emission rates are given normalized per total dried vascular plant biomass. Means of *n* = 9 ± *SE*

**Figure 5:**
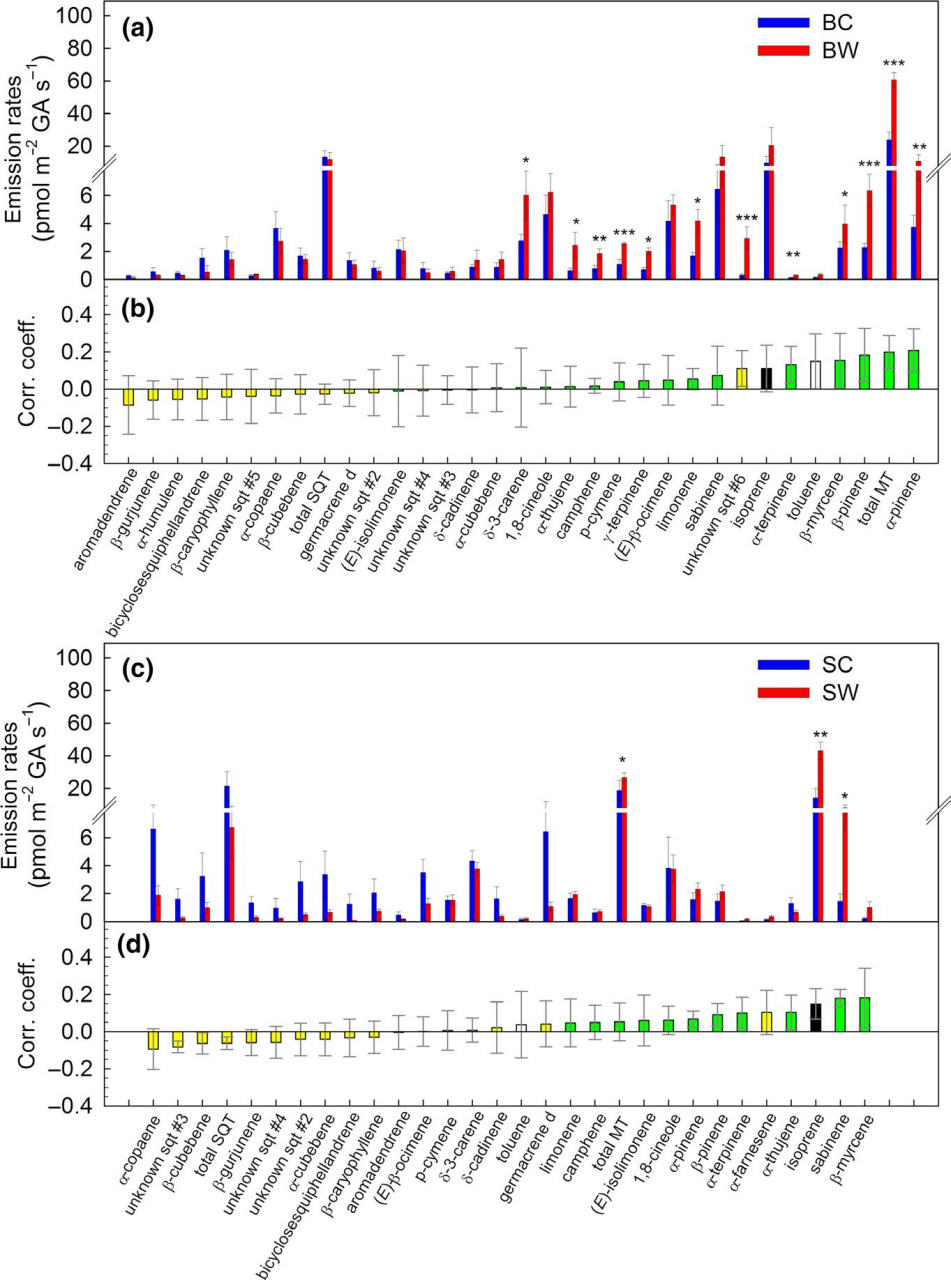
Volatile organic compound (VOC) emissions from (a) “*Betula*” and (c) “*Salix*” mesocosms under control and warming climate conditions. (b, d) Correlation coefficients obtained from orthogonal partial least‐squared discriminant analysis (OPLS‐DA, details given in Figures S6 and S7), showing the correlations between increasing (positive values) and decreasing (negative values) VOC emissions to warming treatment, in “*Betula*” (panel b) and “*Salix*” (panel d) mesocosms. Emission rates are given per ground area (GA). Data were collected in the afternoon (15:30–16:30 CET) during the last ^13^CO_2_‐labeling hour from individual, cuvette‐enclosed mesocosms and analyzed by GC‐MS. Statistical comparison of treatment effect within the mesocosm species: *p < .05, **p < .01, ***p < .001. Means ± *SE* (*n* = 9). The detailed statistical analysis is given in Table S3. B, *Betula*; C, control; S, *Salix*; W, warming. Color code panels (b, d): isoprene (black), monoterpenes (green), sesquiterpenes (yellow), benzenoid (white)

To reliably measure net ecosystem CO_2_ exchange (NEE, Figure 3) and VOC emissions (Figure 4), individual mesocosms were enclosed into small flow‐through cuvettes with a low airflow (Figure S3). Using this setup, we observed diurnal cycles of isoprene, monoterpenes (MT), SQT, methanol, toluene, and lipoxygenase (LOX) products (Figure 4). However, the use of the additional cuvette decreased the temperature differences between C and W treatments from 4.8 ± 0.3 to 3.9 ± 0.1°C during night (23–02 hr) to 3.2 ± 0.1°C during day (13–16 hr; Figure 3a). Regardless of the decrease in temperature difference, we found that warming enhanced almost all VOC emissions (*p* < .001), except for the LOX products and SQT, which significantly (*p* < .01 for LOX) or tended toward reduced emissions (*p* = .056 for SQT; Figures 3 and 4; Figure S5). This observation was consistent for VOC emission rates normalized to total dry plant biomass (foliage plus stem; Figure 4) or to ground‐area (Figure 5; Figure S5). Emissions of total MT, SQT, and toluene (*p* < .05) differed between *Betula* and *Salix* mesocosms, but not for isoprene, methanol, and LOX products (*p* > .05; Figures 4 and 5; Figure S5; Table S3). OPLS‐discriminant analysis showed that emissions of hemiterpene isoprene and the MTs, α‐pinene and β‐pinene, were most associated with the warming treatment in the *Betula* mesocosms, and isoprene and the MTs, sabinene and β‐myrcene, in the *Salix* mesocosms. Emissions of most SQTs were negatively correlated, that is, decreased with warming (Figure 5b,d; Figure S6 and S7). Under warming, the volatile profiles of the two mesocosm types strongly differed: Emissions of isoprene and the SQTs δ‐elemene and α‐selinene were characteristic for *Salix* mesocosms, whereas emissions of most of the MTs and the benzenoid toluene characterized *Betula* mesocosms (Figure S8).

### 3.2 ^13^C‐incorporation into VOC

**Figure 6:**
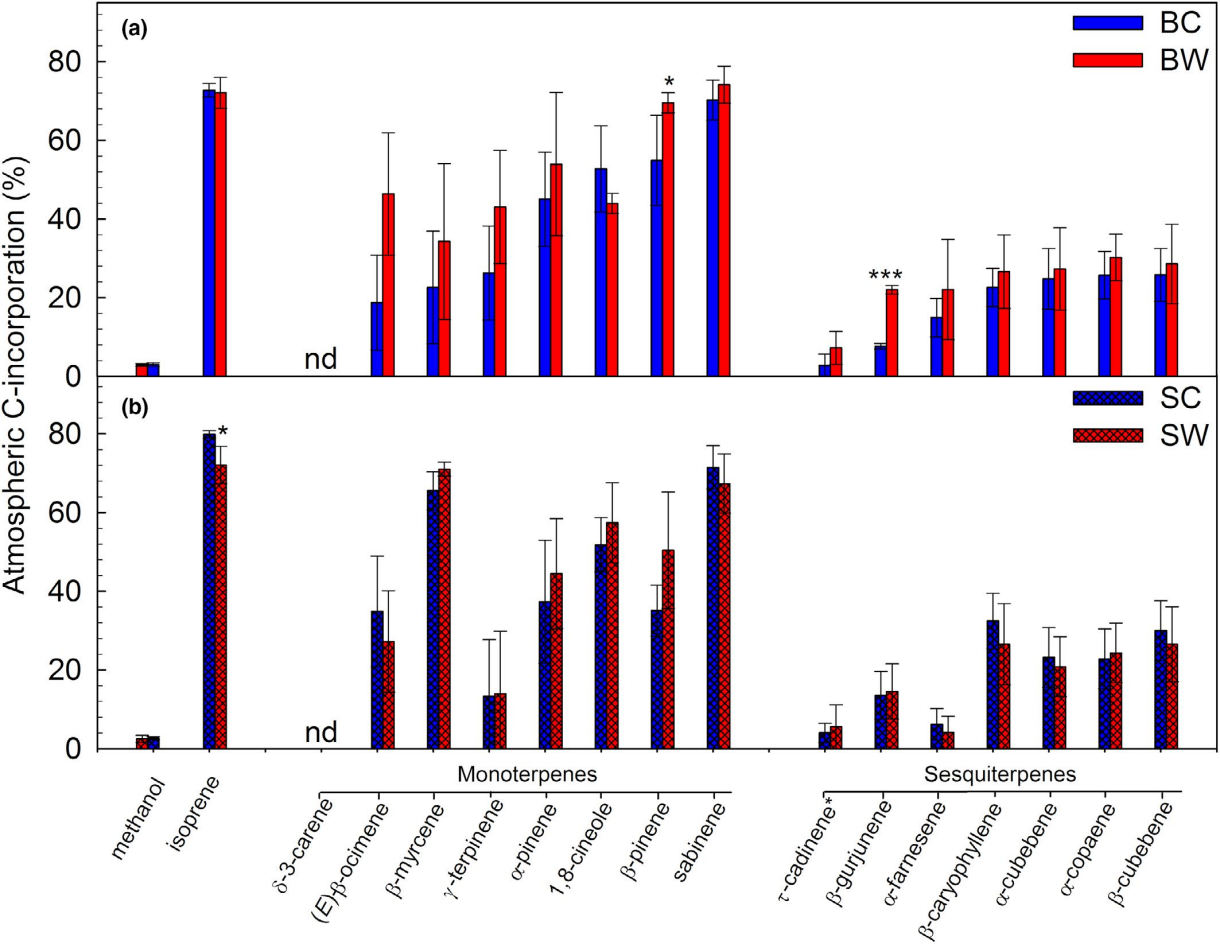
Incorporation of atmospheric C into methanol, isoprene, and the top‐five most emitted monoterpenes and sesquiterpenes from (a) “*Betula*” and (b) “*Salix*” mesocosms, respectively. Data depict the 30 min average at 4.5 hr from starting the ^13^CO_2_‐labeling. Data were collected from individual, cuvette‐enclosed mesocosms and except for methanol, originated from GC‐MS measurements. Data were normalized to 100% of ^13^CO_2_‐labeling gas, after estimation of ^13^CO_2_ concentrations inside the cuvettes (see Section 2). Statistical significance was tested with ANOVA and Holm–Sidak method for pairwise multiple comparison procedures. Comparison for factor: C versus W within mesocosm (*). B, *Betula*; C, control; S, *Salix*; W, warming; *p < .05; ***p < .001. Means ± *SE* (*n* = 6); nd, nondetectable

We used a ^13^C‐labeling technique to trace the carbon atoms from atmospheric CO_2_ through the plant fixation process until its re‐emission as VOCs. For several VOCs, the incorporation of atmospheric C after 4.5 hr of ^13^C‐labeling was found to be significant compared to ^12^CO_2_ control experiments (*p* < .001, paired *t* test; Figures S9 and S10). Among the most emitted VOCs, isoprene exhibited the closest link to photosynthesis and methanol the furthest, with 72%–80% and 2.5%–2.9% incorporation of atmospheric CO_2_ into isoprene and methanol, respectively (Figure 6; Figures S9 and S10). Overall, the incorporation of ^13^C into volatile terpenoids decreased from 72%–80% of the C_5_ isoprene to 33%–46% of the C_10_ monoterpenes and to 18%–26% of the C_15_ sesquiterpenes. These significant decreases (ANOVA, *p* < .001) appeared to be linked to the number of C contained: the unlabeled (^12^C) portion per atom of carbon was similar, 4%–6.7% C^−1^ across all terpenoids.

GCMS analysis of the different MT and SQT isomers showed that the incorporation of atmospheric CO_2_ into terpene biosynthesis was highly compound‐specific (Figure 6; Figures S9 and S10). For instance, δ‐3‐carene was not significantly labeled among the top‐five most emitted MTs, whereas β‐pinene and sabinene were 35% and 74% labeled, respectively. Notably, the incorporation of ^13^C was affected by warming treatment (*p* < .001), as well as mesocosm type (*p* < .05). Interestingly, incorporation of atmospheric CO_2_ into isoprene decreased under warming in *Salix* mesocosms, while that of MTs increased in *Betula* mesocosms. We observed a weak (0.63 ± 0.18%), but significant ^13^C‐label in toluene (*p* < .001).

### 3.3 De novo biosynthesis of monoterpenes is of great importance in subarctic heath tundra

The rapid incorporation of atmospheric ^13^CO_2_ into emitted monoterpenes demonstrates that MT biosynthesis is active in subarctic mesocosms, but it does not give alone the portion of de novo and pool emissions required to improve modeling (Ghirardo et al., 2010). Therefore, we calculated the de novo MT biosynthesis based on the ^13^C‐labeling and isoprene measurements.

**Figure 7:**
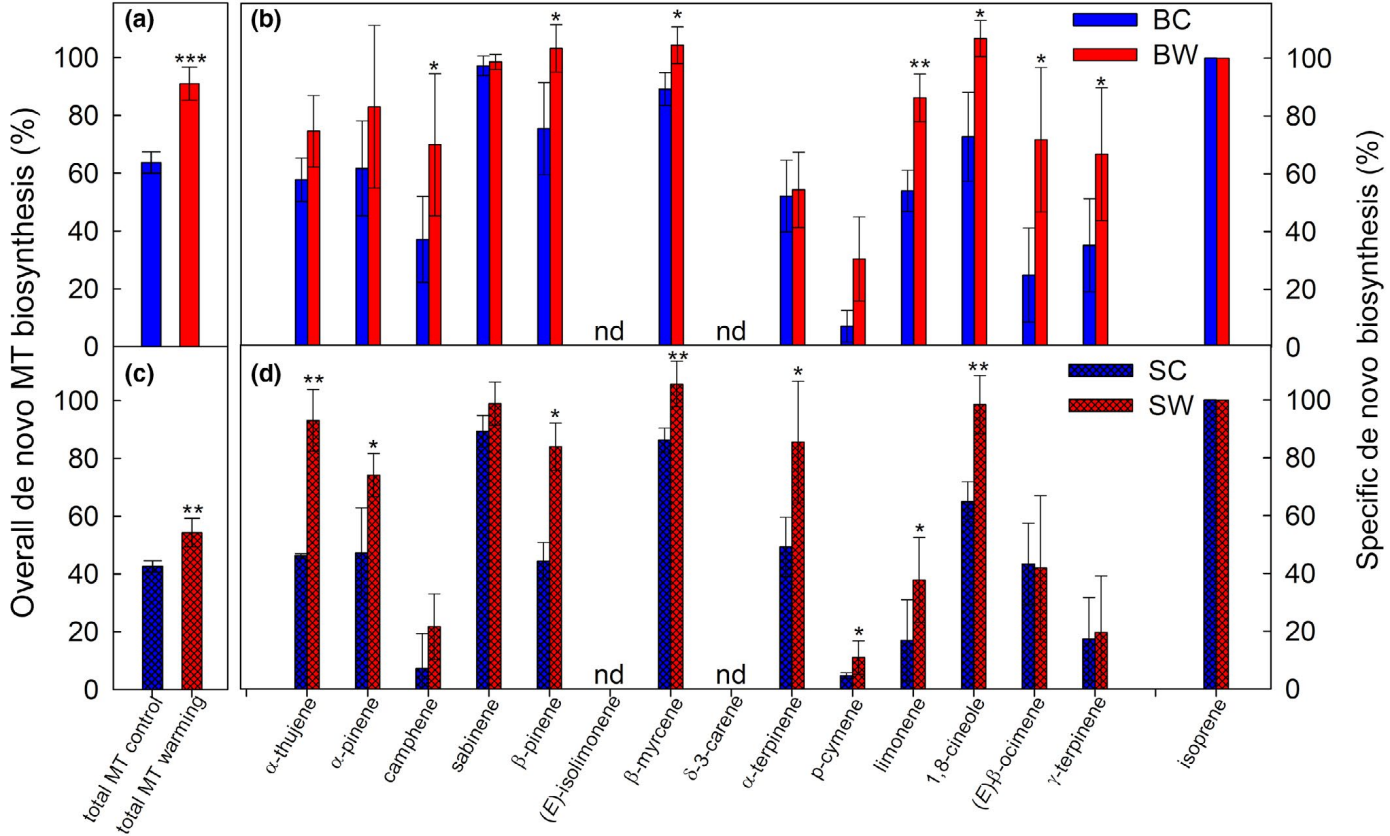
Contribution of de novo monoterpene biosynthesis for *Betula* (B) and *Salix* (S) mesocosms under control (C) and warming (W) climates. (a, c) Total de novo monoterpene biosynthesis obtained from the whole spectra of emitted monoterpenes; data originated from PTR‐ToF‐MS measurements. (b, d) Specific de novo biosynthesis of monoterpenes compared to isoprene, which was set to 100% as its biosynthesis is always de novo (Ghirardo et al., 2010); data originated from GC‐MS measurements of cuvette‐enclosed, individual mesocosms. Plots: (a, b) refer to “*Betula*” and (c, d) to “*Salix*” mesocosms under control (C, in blue) and warming (W, in red) conditions. *p < .05; **p < .01; ***p < .001. Means ± *SE* (*n* = 6); nd, nondetectable de novo MT biosynthesis, refer to those compounds emitted without significant ^13^C‐incorporation

We observed that the total MT emissions from subarctic mesocosms were largely derived from de novo biosynthesis (Figure 7a,c). In the actual climate, the de novo production was significantly different between *Betula* (64%) and *Salix* (43%; *p* < .001). These partitions significantly increased under warming to 91% and 54% in *Betula* and *Salix* mesocosms, respectively (*p* < .001). Overall, the effects of treatment and vegetation type on de novo biosynthesis were also seen on individual MT isomers (warming effect, *p* < .001; mesocosm effect, *p* = .002). Warming strongly increased the de novo versus pool emissions of some, but not all, monoterpenes (Figure 7b,d).

Consistently, emission rates of monoterpenes showing a conspicuous de novo synthesis were differently emitted in the morning and in the afternoon under the same air temperature (i.e., same pool emissions; Figure 5; Figure S5; Table S3) for *Betula‐* and *Salix*‐based mesocosms, reflecting the typical diurnal variation expected from MT biosynthesis (see also Figure 4c,d). Notably, and independent of the treatment effect, the origin of specific MTs strongly differed: among relevantly emitted MTs (Table S3), (*E*)‐isolimonene, and δ‐3‐carene were completely light‐independent, whereas sabinene (*Betula*) and β‐myrcene (*Salix*) were mainly light‐dependent (86%–100%, Figure 7). Overall, de novo biosynthesis calculated from GC‐MS and PTR‐ToF‐MS data matched well (*R* ^2^ = .988 and .967 for MT and SQT, respectively; see Figure S11). Taken together, the high de novo percentage indicates that factors affecting the biochemical processes involved in MT biosynthesis are crucial in controlling the MT emissions from subarctic heath tundra. Nevertheless, temperature alone, can also impact the pool emissions, comprising a significant portion (9%–57%) of total monoterpene emissions.

### 3.4 Above‐ and belowground C‐allocation

**Figure 8:**
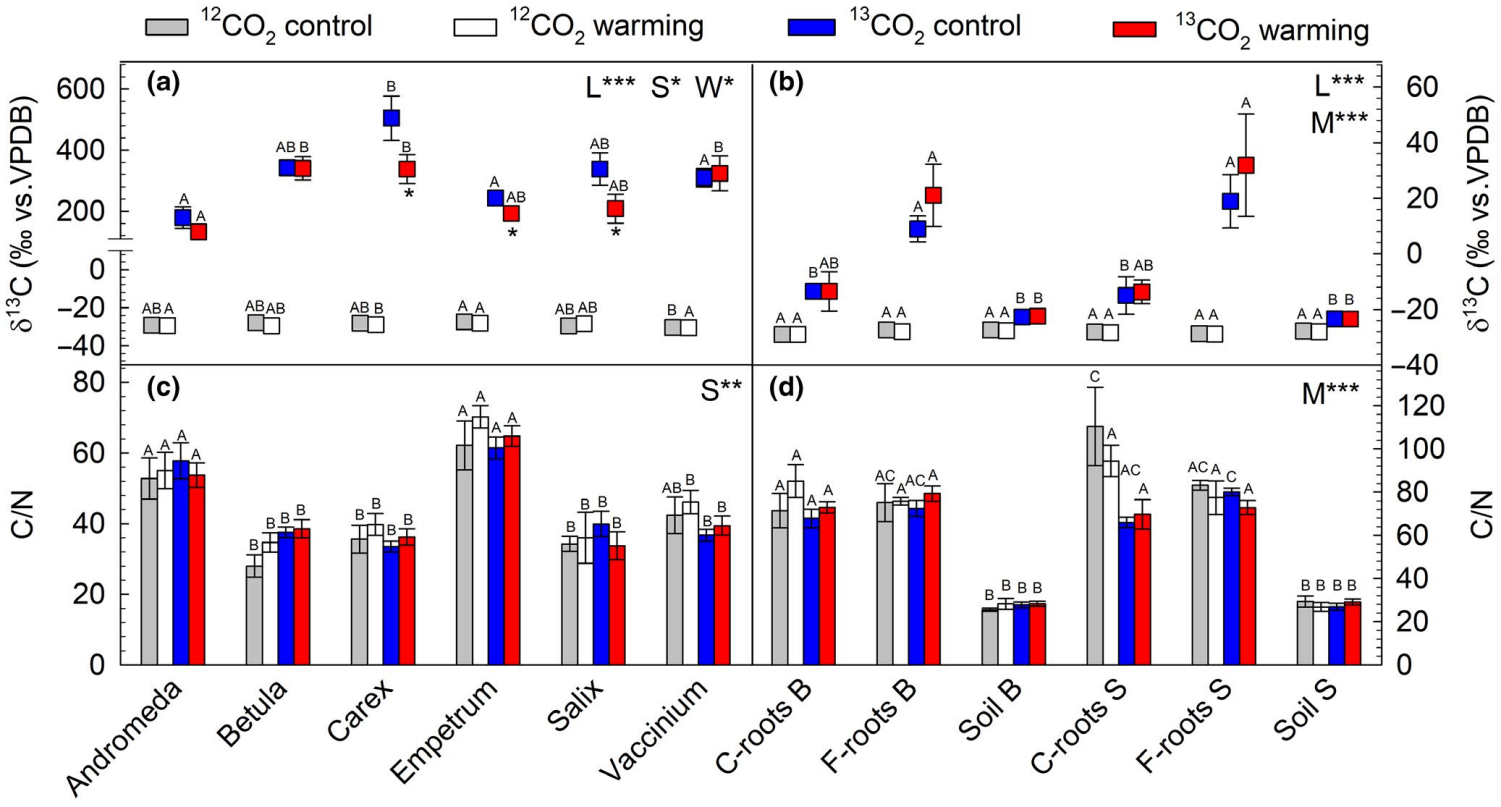
(a, b) Isotopic signature of δ^13^C and (c, d) C/N ratios in aboveground (left panels a, c) plant material (leaves) from the six most abundant plant species (*Andromeda polifolia*, *Betula nana*, *Carex* spp.,* Empetrum nigrum* ssp.* hermaphroditum*, *Salix myrsinites*, and *Vaccinium uligonosum*) and in belowground (right panels b, d) coarse roots (C‐roots), fine roots (F‐roots), and soil from the two mesocosm types, “*Betula*” (B) and “*Salix”* (S). Statistical main effects (L, labeling; M, plant/soil material; S, plant species; W, warming) are reported in panel; statistically significant differences between plant species (a, c), and belowground compartments (b, d) within the treatments (^12^CO_2_ control, ^12^CO_2_ warming, ^13^CO_2_ control, ^13^CO_2_ warming) are reported above bars using different letters (*p* < .05); asterisk (*) below the red squares indicates significant warming effect at *p* < .05 under ^13^CO_2_ labeling within the plant species. Means ± *SE* (*n* = 6)

To understand subarctic ecosystem responses to global warming, we studied the allocation of carbon in different plant tissues, soil, and microbial biomass. Compared to the control climate, warming mainly affected the C‐allocation of aboveground tissues, as seen by the significant (*p* < .05) decreases in the δ^13^C signature found in *Carex*, *Empetrum*, and *Salix* under ^13^CO_2_‐labeling, whereas the C‐allocation in belowground plant tissues and soil remained unchanged (*p* > .05; Figure 8a,b). This is in line with the lower NEE observed under the warming climate (*p* < .05; see Figure 3 for NEE analysis). Isotope analyses of ^12/13^C in the different plant species indicated that the most photosynthetically active species were *Carex*, *Betula*, and *Salix*, as seen by their highest enrichment of ^13^C after ^13^CO_2_‐labeling. The δ^13^C signature of *Carex* was significantly higher than that of *Andromeda*, *Empetrum*, and *Vaccinium* under the control climate, and higher than for *Andromeda* under the warming climate (Figure 8a). Under the ^12^CO_2_ atmosphere, the δ^13^C signature was −28.9 ± 0.3‰, which is in good agreement with the field studies (Ravn, Ambus, et al., 2017).

Belowground, the ^12/13^C signatures of fine roots, coarse roots, and soil were significantly and differently enriched in ^13^C (*p* < .001) compared to control samples collected under a ^12^CO_2_ atmosphere (−28.2 ± 0.2‰; Figure 8b). The highest significant enrichments in ^13^C were found in fine roots, followed by coarse roots and soil, roughly indicating the translocation of C from freshly assimilated carbons within the foliage toward C‐allocation throughout the entire mesocosm. Notably, the significant decreases (*p* < .05) of C‐allocation in aboveground tissues were not balanced by concomitant increases in the δ^13^C signature of fine and coarse roots under a warming climate (*p* = .14). This observation indicates a minor shift in C‐allocation toward belowground tissues under warming. In line with this, we did not find appreciable changes of ^13^C‐fluxes dependent on mesocosm or warming when we considered all the plant species composing the mesocosm together (Figure S12). Therefore, warming caused an overall lower C allocation in aboveground tissues, mainly due to the lower NEE and higher VOC emissions (Figure S13). Our data on ^13^C‐labeling of VOCs indicates a substantial allocation of C into volatiles (8%–11% of NEE), which increased to 15%–33% under warming.

**Figure 9:**
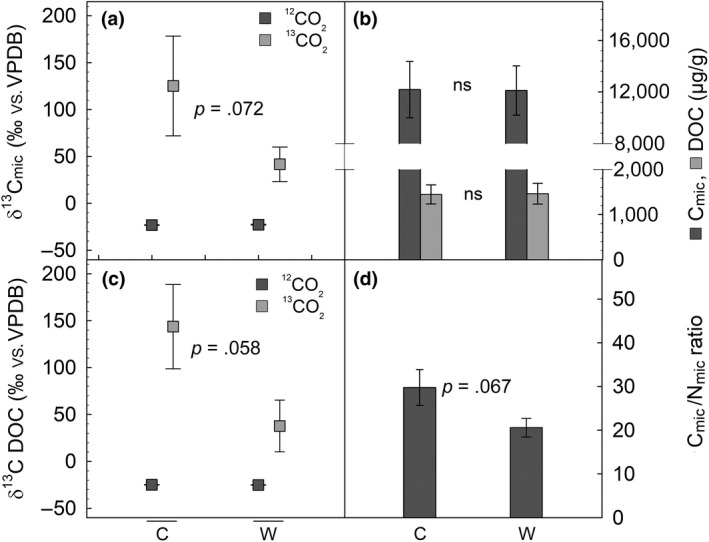
Isotopic signatures (δ^13^C) of the (a) microbiome carbon (C_mic_) and (c) the dissolved organic carbon (DOC); total levels of (b) C_mic_ and DOC; carbon and nitrogen ratios of the microbiome (C_mic_/N_mic_; d) under control (C) and warming (W) climate. The mesocosm types “*Betula*” and “*Salix*” have been pooled together. Statistical differences between control and warming were tested by one‐way ANOVA. The ^13^C enrichment of samples under a ^13^CO_2_ atmosphere was statistically significant compared to those under ^12^CO_2_ (*p* < .001). Means of *n* = 3–9 ± *SE*. ns, not significant

To understand whether the lower NEE under warming climate negatively affected the soil microbiome and other organic matter present in the soil, we investigated, in detail, the ^13^C enrichment of different soil C pools. Microbial carbon (C_mic_) and DOC were highly enriched in ^13^C after the 5.5 hr ^13^CO_2_ labeling compared to ^12^CO_2_ controls (*p* < .001, Figure 9a,c), indicating a fast translocation of freshly assimilated carbon to soil. Warming climate tended to lower ^13^C enrichment of C_mic_ (*p* = .072) and DOC (*p* = .058) compared to the actual climate. The overall C_mic_ and DOC did not differ between mesocosms or change after more than 1 month of warming treatments (Figure 9b). Aboveground, the C/N ratio was plant species specific, but did not change significantly under warming either in the aboveground or belowground plant tissues (*p* > .05; Figure 8c,d). However, warming appeared to decrease the C/N ratio of the microbiome (*p* = .067; Figure 9d).

## 4 DISCUSSION

### 4.1 Warmer subarctic climate increases volatile emissions

The present study, involving climate simulation chambers, allowed us to investigate the influence of current and predicted warmer climates on the emission of VOCs from subarctic ecosystems. Our data show that warming leads to a significant increase of several volatiles, including terpenoid, alcohol, and benzenoid compounds (*p* < .01, ANOVA; Figures 2, 3, 4, 5). Increasing monoterpene and isoprene emissions are consistent with findings from recent field studies of arctic tundra vegetation (Faubert et al., 2010; Kramshøj et al., 2016; Lindwall, Schollert, et al., 2016; Lindwall, Svendsen, et al., 2016; Valolahti et al., 2015) and to general expectations (Peñuelas & Staudt, 2010 and reference therein). However, field studies could not distinguish to what extent the warming impacted terpenoid emissions by changing the plant physiological processes underlying the emissions (Kramshøj et al., 2016) or by affecting the biomass and species composition of plants (Valolahti et al., 2015). Here we demonstrate that regardless of the composition of the plant species and changes in plant biomass (see emission rates normalized per leaf biomass in Figure 4), terpenoid emissions will increase under a warmer climate. Such direct temperature effects are also supported by measurements from Disko Island, Greenland, where a short‐term increase in VOC emissions in response to warming was observed before changes in vegetation were detectable (Lindwall, Schollert, et al., 2016; Lindwall, Svendsen, et al., 2016). Compared to these studies, the temperature effects observed here are less pronounced. This is most likely due to the constant airflow through the phytotron chambers. Our experimental design enabled us to maintain the +5°C temperature difference without introducing confounding factors, such as wind or changes in humidity in the plant canopy between the treatments, which are typically altered in simulated warming treatments using open‐top chambers. Nevertheless, the present study focused on the mechanisms involved in VOC emissions. Studies under controlled environmental conditions, such as this one, are paramount to gain a mechanistic understanding of the processes controlling terpene emissions, necessary for the correct projection of future VOC emissions from the Arctic (see next section).

Our study shows, for the first time, that the chemical pattern of ecosystem VOC emissions in the subarctic region is dependent on plant species composition (Figures 2, 4 and 5; Tables S1 and S3). From our laboratory results, we expect that global warming will significantly increase MT emissions from heath tundra dominated by *Betula* spp. and isoprene emission from *Salix* spp.‐dominated vegetation communities. This corresponds to the typical VOC emission pattern of these two plant species (Ghirardo et al., 2016; Kellomäki, Rouvinen, Peltola, Strandman, & Steinbrecher, 2001; Tarvainen, Hakola, Rinne, Hellén, & Haapanala, 2007; Vedel‐Petersen, Schollert, Nymand, & Rinnan, 2015). Surprisingly, our results indicate that warming can lead to a decrease in the emission of SQTs, especially in vegetation dominated by *Salix* (*p* < .05). Decreasing plant SQT emissions at higher temperatures are generally not expected (e.g., Staudt & Lhoutellier, 2011), although increasing isoprene/monoterpene emissions could lead to a lower availability of biochemical precursors for SQT biosynthesis, as common intermediates are imported from the cytosol into the plastids (Hemmerlin et al., 2003; Mendoza‐Poudereux et al., 2015). Another explanation to the decrease of SQT emissions in subarctic vegetation with warming is that a striking portion of SQT emissions originate from soil microorganisms (e.g., Bourtsoukidis et al., 2018; Peñuelas et al., 2013; Weikl et al., 2016), which change their activity pattern as a result of higher temperatures.

For chemical processes in the lower atmosphere, changes in the chemical composition of VOCs, as well as changes in emission rates, are crucial, since the rate constant for the gas phase reaction between the various VOCs with ozone, hydroxyl, and nitrate differs to a large extent (e.g., Fuentes et al., 2000). Our study not only shows that warming increases the input of VOCs from the subarctic tundra into the atmosphere, but also that warming leads to a shift from less reactive (e.g., methanol) toward very reactive (e.g., isoprene, MTs) hydrocarbons, which is important for atmospheric chemistry (Figure 2). For example, the methanol‐to‐isoprene carbon ratio decreased from 3.0 (ambient) to 1.4 (warming; Figure S4). On the other hand, we observed declining emissions of the highly reactive SQTs. Such changes in chemical composition and loads into the atmosphere can influence the formation of secondary organic aerosols and photochemical ozone production (Atkinson & Arey, 2003). Because the impact of terpenoids in chemical communication strongly depends on the VOC blend, future changes in terpenoid compositions may lead to alteration of plant‐to‐plant (Riedlmeier et al., 2017) and plant‐to‐insect/microbe interactions (Ghirardo et al., 2012; Pichersky & Gershenzon, 2002), as well as affecting the herbivory pressure at the ecosystem level (Heil, 2008).

### 4.2 The origin of monoterpene emissions and implications for the VOC modeling approach

We labeled mesocosms with ^13^CO_2_ to study the biochemical origin of the terpenoid emissions from subarctic ecosystems and to quantify the proportion of “de novo” versus “pool” emissions (Ghirardo et al., 2010). Isoprene and monoterpene emissions from direct de novo biosynthesis are generally called “light‐dependent” emissions because their production relies on photosynthesis and follows light‐ and temperature‐dependent processes (Loreto et al., 1996; Loreto & Schnitzler, 2010). The evaporation of MTs from internal and external resin storage, as well as from nonspecific storage pools (Delfine, Csiky, Seufert, & Loreto, 2000; Loreto et al., 2000; Noe, Ciccioli, Brancaleoni, Loreto, & Niinemets, 2006) however, is mainly under the control of temperature, which physically forces the volatile compounds to escape their storage structures by affecting the evaporation processes (Grote & Niinemets, 2008).

The correct mathematical description of isoprene and MT emissions from de novo biosynthesis in emission algorithms is essential for reliable modeling of VOC emissions regionally and globally (e.g., Grote et al., 2014; Guenther et al., 2012). As an important result, we showed that the de novo biosynthesis of MTs makes a significant contribution to the total emissions of these compounds from subarctic heath tundra. Furthermore, we showed that the share of emissions from de novo processes and evaporation from pools highly depends on the species composition of the vegetation communities and changes with global warming. Under ambient climatic conditions, both emission sources—de novo and pools—are of approximately similar importance, although the proportion of freshly synthesized monoterpenes in *Salix* mesocosms was lower at 40%–44% compared to 60%–68% in *Betula* ones (Figure 7). Warming increased the share of de novo sources to 50%–58% in *Salix*‐dominated mesocosms and to 87%–95% in *Betula*‐dominated mesocosms. Within the genus *Betula*, the species *B. pendula* and *B. pubescens* are the most studied. Both species are described as strong MT emitters (Hakola et al., 2001) and the emissions solely originate from de novo biosynthesis (Ghirardo et al., 2010). Therefore, it is logical to assume that *B. nana*, also a MT emitter (Vedel‐Petersen et al., 2015), has no special structures for the storage of MTs in the leaves. On the other hand, the trees and shrubs in the genus *Salix* are described as very strong isoprene emitters (Isebrands et al., 1999; Kesselmeier & Staudt, 1999; Vedel‐Petersen et al., 2015), although low MT emissions have also been observed in the same genus (e.g., *Salix babylonica*, Ghirardo et al., 2016; *Salix arctophila*, Vedel‐Petersen et al., 2015).

Our measurements demonstrate that the ^13^C‐label was incorporated to varying degrees into the individual MTs, suggesting different emission sources (Figure 6). For δ‐3‐carene, for example, no incorporation of ^13^C into the molecular structure was detectable, suggesting that the emitted δ‐3‐carene was not biosynthesized in the chloroplasts during ^13^CO_2_‐feeding, but that the biosynthesis of this molecule had occurred earlier. The observed emission can therefore be attributed to the evaporation of δ‐3‐carene from storage organs, likely in the evergreen species, which dominated the mesocosms (Kesselmeier & Staudt, 1999). This purely thermodynamic process often occurs in evergreen plant species. An earlier ^13^CO_2_‐labeling study with *Pinus ponderosa* (Harley et al., 2014) also described low (9%) partition of δ‐3‐carene in light‐dependent emissions, which were dominated by other MTs such as sabinene (74%) and myrcene (90%). This is similar to our study, where sabinene and myrcene had high incorporation rates of ^13^C, and similar to isoprene, their emissions were entirely light‐dependent (Figures 6 and 7). Alternate emission sources for non‐^13^C‐labeled MTs from mesocosms include the biological activity of bacteria and fungi in soils and the decomposition of leaf litter and soil organic matter (Gray, Monson, & Fierer, 2010; Guo et al., 2019; Peñuelas et al., 2014). Monoterpenes are released from decomposing litter of arctic *Salix* spp. and in higher amounts from evergreen shrub litter with storage reserves (Svendsen et al., 2018).

In summary, we show the importance of de novo processes in MT emissions from subarctic tundra. Since some environmental dependencies (e.g., light, CO_2_) affect the de novo biosynthesis of MTs to different degrees in the different compartments of the ecosystem, modeling approaches must take into account both de novo and pool emissions (Ghirardo et al., 2010, 2016). Furthermore, warming appears to increase the share of de novo synthesized emissions compared to emissions from storage. The canopy of the subarctic heath tundra has fine‐scale variation within many species, which makes the determination of species‐specific VOC emissions difficult. For the accurate estimation of current and future VOC emissions from this highly climate‐sensitive ecosystem, it seems therefore more appropriate to parameterize the modeling based on ecosystem data.

### 4.3 Impact of climate warming on C‐allocation within plant–soil subarctic ecosystems

In the terrestrial carbon cycle, we still lack understanding of the fate of freshly assimilated C allocated within plants and soil, and the losses (e.g., as CO_2_/CH_4_ or VOC emissions into the atmosphere; Brüggemann et al., 2011). In the current study, the use of ^13^CO_2_ allowed us to track the atmospheric carbon to assess the effects of climate warming on the C cycle and vegetation communities of the subarctic heath tundra (Figures 8 and 9).

The decreasing ^13^C isotopic enrichment levels from aboveground tissue to the root system, microorganisms, and soil showed that atmospheric carbon is transferred to the rhizosphere within a few hours. The belowground C‐allocation is a conspicuous part of the C fixed by plants (up to 40%; Kuzyakov, 2002). Here a portion of C is stored and respired by the roots, while another part of the C is released as root exudates into the rhizosphere, where it is available to the soil microorganisms and becomes part of the soil organic matter that can, in turn, be lost by heterotrophic respiration (Pausch & Kuzyakov, 2018; Ruehr et al., 2009).

In our study, warming led to a reduction in total C‐sequestration by reducing NEE in the tundra vegetation. Hence, warming stimulated ecosystem respiration more than photosynthesis. Long‐term studies of Canadian high arctic and of subalpine meadow have shown increasing net primary production (NPP) under current warming (Harte, Saleska, & Levy, 2015; Hudson & Henry, 2009). Since NEE equals NPP minus heterotrophic respiration, we assume that the lowered NEE under the warmer climate conditions was caused mainly by higher soil respiration rates. In fact, our measurements indicate a higher value of soil respiration, as much lower NEE values (especially in the *Salix* spp. dominated mesocosms) were observed under warming at low radiation intensities (nights and predawn) compared to daytime conditions (Figure 3). The higher heterotrophic soil respiration under the subarctic warming scenario is consistent with convincing results from broader recent studies showing that soil respiration is currently rising worldwide (Bond‐Lamberty, Bailey, Chen, Gough, & Vargas, 2018) and that the effects of warming on C‐losses from soil carbon stocks may be considerable in high‐latitude areas (Crowther et al., 2016).

Another recent study including field observations and a broad meta‐analysis has highlighted a general change in plant species composition, despite unchanged NPP, in response to climate change, which caused a shift from aboveground to belowground productivity (Liu et al., 2018). The use of the stable ^13^C isotope in our phytotron studies revealed that in the short term, climate warming impacted the C allocation of some, but not all plant species (i.e., *Carex*, *Empetrum* and *Salix*, but not *Andromeda*, *Betula,* and *Vaccinium*). Although most C appeared to be more retained in the fine roots, a fraction was rapidly exported into soil and used by soil microorganisms. In turn, such changes of plant species‐specific C allocation and translocation have the potential to alter future plant species compositions of subarctic ecosystems, as was recently postulated (Valolahti et al., 2015).

Our data suggest that under a warming climate, the C‐allocation belowground is stimulated within the plants, as seen by the lower ^13^C in DOC and microbes and higher amount of ^13^C in fine roots. However, such changes in belowground processes seem to occur very slowly. Under our warming treatment, temperature and the weak change of C partitioning belowground did not affect the total microbial biomass carbon pool. This agrees with previous field observations showing that more than 10 years are needed to develop significant changes in microbial biomass and community composition in the arctic heaths (Rinnan et al., 2007). Overall, our analysis shows the dynamics of C‐allocation within the plant–soil system and indicates negative effects of climate warming on C‐sequestration in subarctic ecosystems that may cause shifts in vegetation compositions, microbial communities, and soil organic matter in the long term.

## Supporting information

Supinfo
