# Supplementary material for: Origin of volatile organic compound emissions from subarctic tundra under global warming
Source: Glob Chang Biol. 2020 Jan 20;26(3):1908–25. doi: 10.1111/gcb.14935 (PMC7078956; doi:10.1111/gcb.14935)

**Supporting Information of:**

**Origin of VOC emissions from subarctic ecosystems under global warming**

Andrea Ghirardo, Frida Lindstein, Kerstin Koch, Franz Buegger, Michael Schloter, Andreas Albert, Anders Michelsen, Jana Barbro Winkler, Jörg-Peter Schnitzler and Riikka Rinnan

***Table of Contents***

Supporting Figures:

**Figure S1**

**Figure S2**

**Figure S3**

**Figure S4**

**Figure S5**

**Figure S6**

**Figure S7**

**Figure S8**

**Figure S9**

**Figure S10**

**Figure S11**

**Figure S12**

**Figure S13**

Supporting Tables:

**Table S1**

**Table S2**

**Table S3**

**
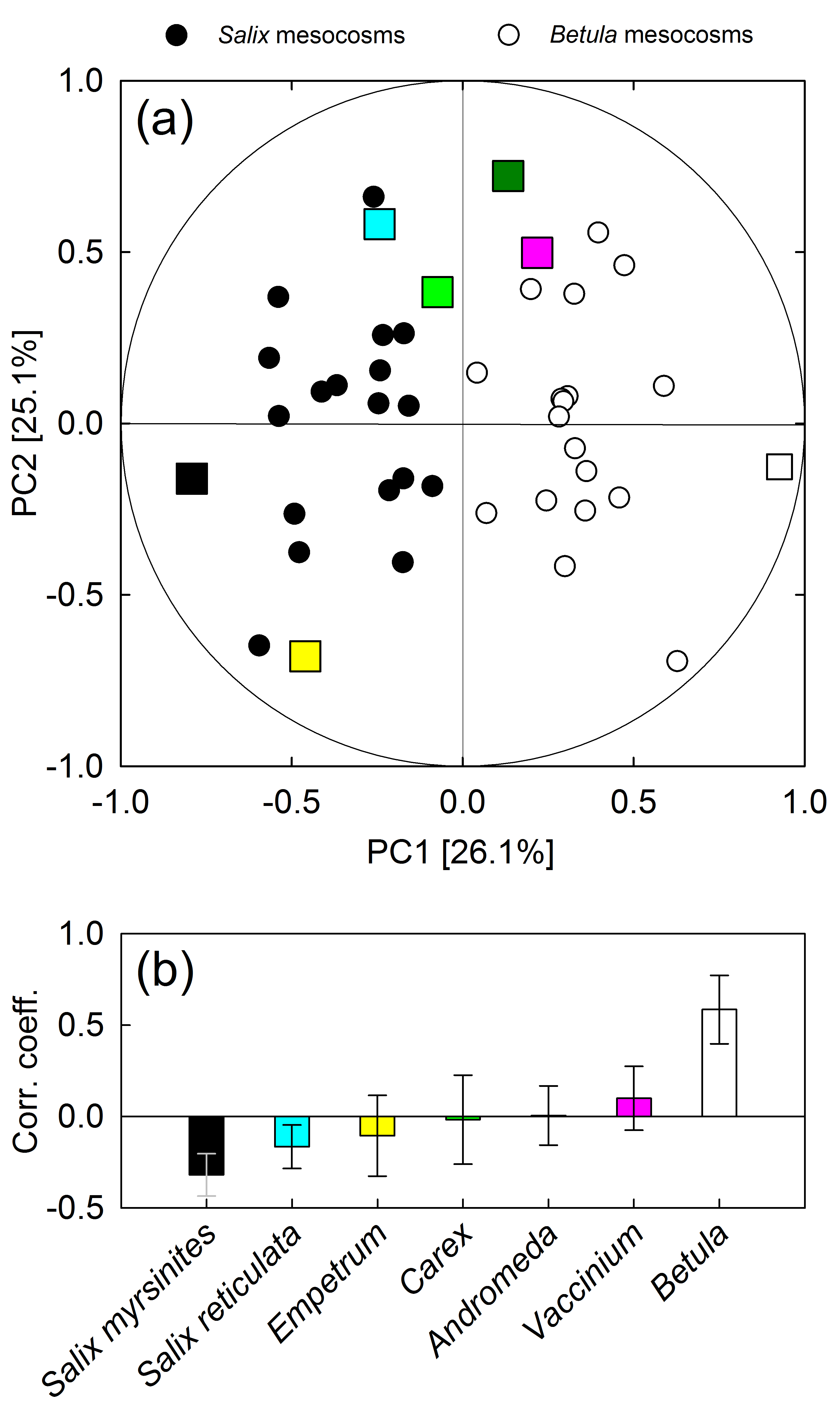
**

**Figure S1. Differences in plant composition in ‘*Salix*’ and ‘*Betula*’ dominated mesocosms. a)** Orthogonal partial least square regression (OPLS) biplot (scores and loading; correlation scaled) of the aboveground plant biomass (foliage plus stem; X-loading, in g dw) from ‘*Salix*’(black circles) and ‘*Betula*’ (white circles) mesocosms. Y-variables: ‘*Salix*’=0, ‘*Betula*’=1. Scores and loading data are depicted with circles and rectangles, respectively. The ellipse indicates the model tolerance based on Hotelling’s *T*^2^ and significance level of α = 0.05. **b**) Correlation coefficient plot of OPLS, correlating biomass data with mesocosms types. The coefficients are given scaled and centered. Error bars were derived using the jack-knife method. Bars represent the average ± se of 18 mesocosms. OPLS model fitness: *r*^2^ (x)=51%, *r*^2^=82%, *q*^2^ (cum)=77% using 1 predictive component. RMSEE (root mean square error of estimation) = 0.22; RMSEcv (root mean square error of cross-validation) = 0.23; *P* = 1.24e^-9^, CV-ANOVA. PC, predictive component. Color code of square symbols in panel (a) reflects those given in panel (b). A list of plant names and biomass data are given in Supp. Table S1.

**
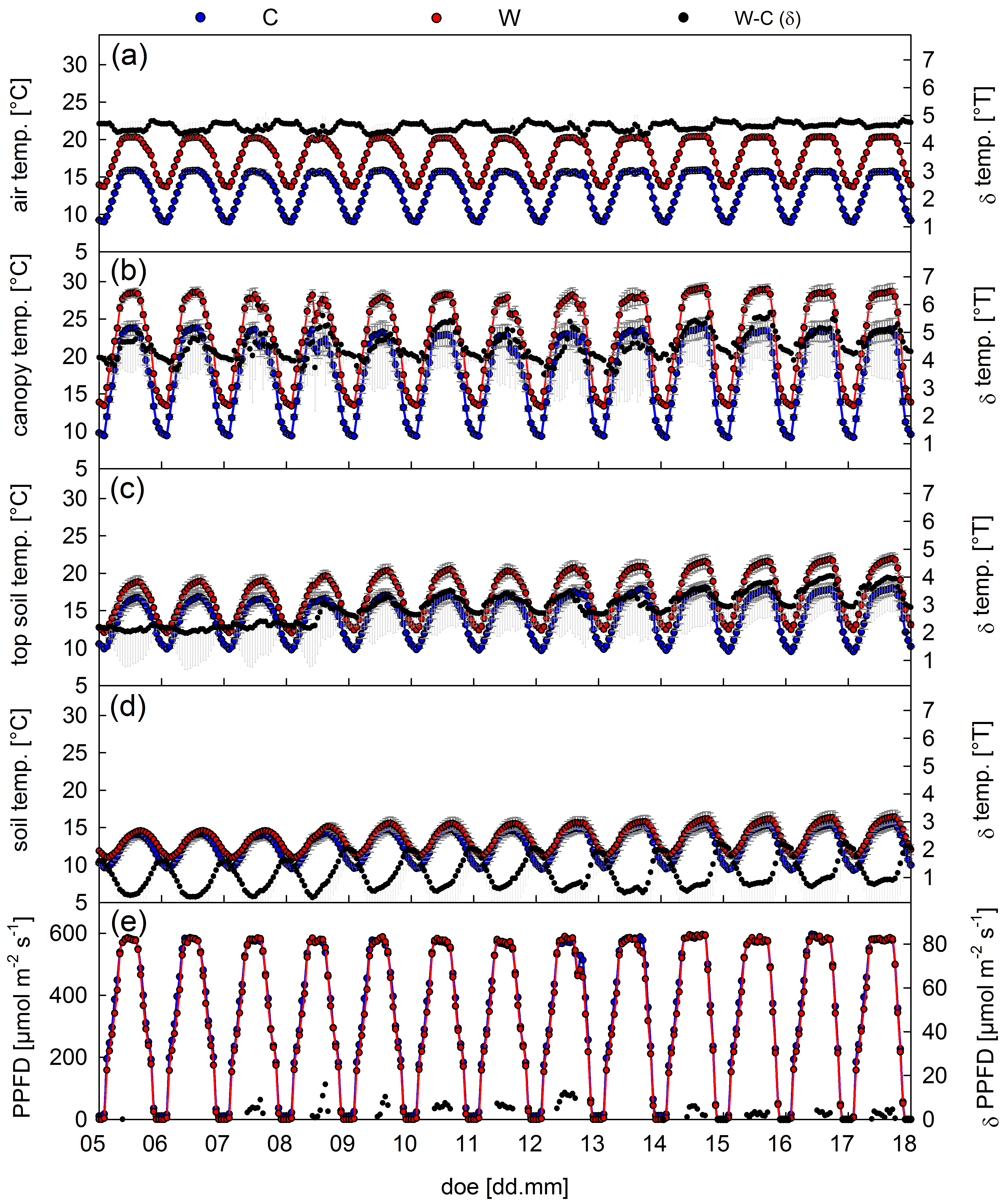
**

**Figure S2. Climate simulation.** Environmental conditions inside the chambers for the control (C, in blue) and warming (W, red) climate simulations. Temperatures of **a**) air, **b**) canopy, **c**) top soil at 0-to-2 cm in depth, **d**) soil at 2-to-5 cm in depth, **e**) light. Absolute differences between control and warming climate parameters are referred as delta (δ, in black). Abbr.: doe, day of experiment.

**
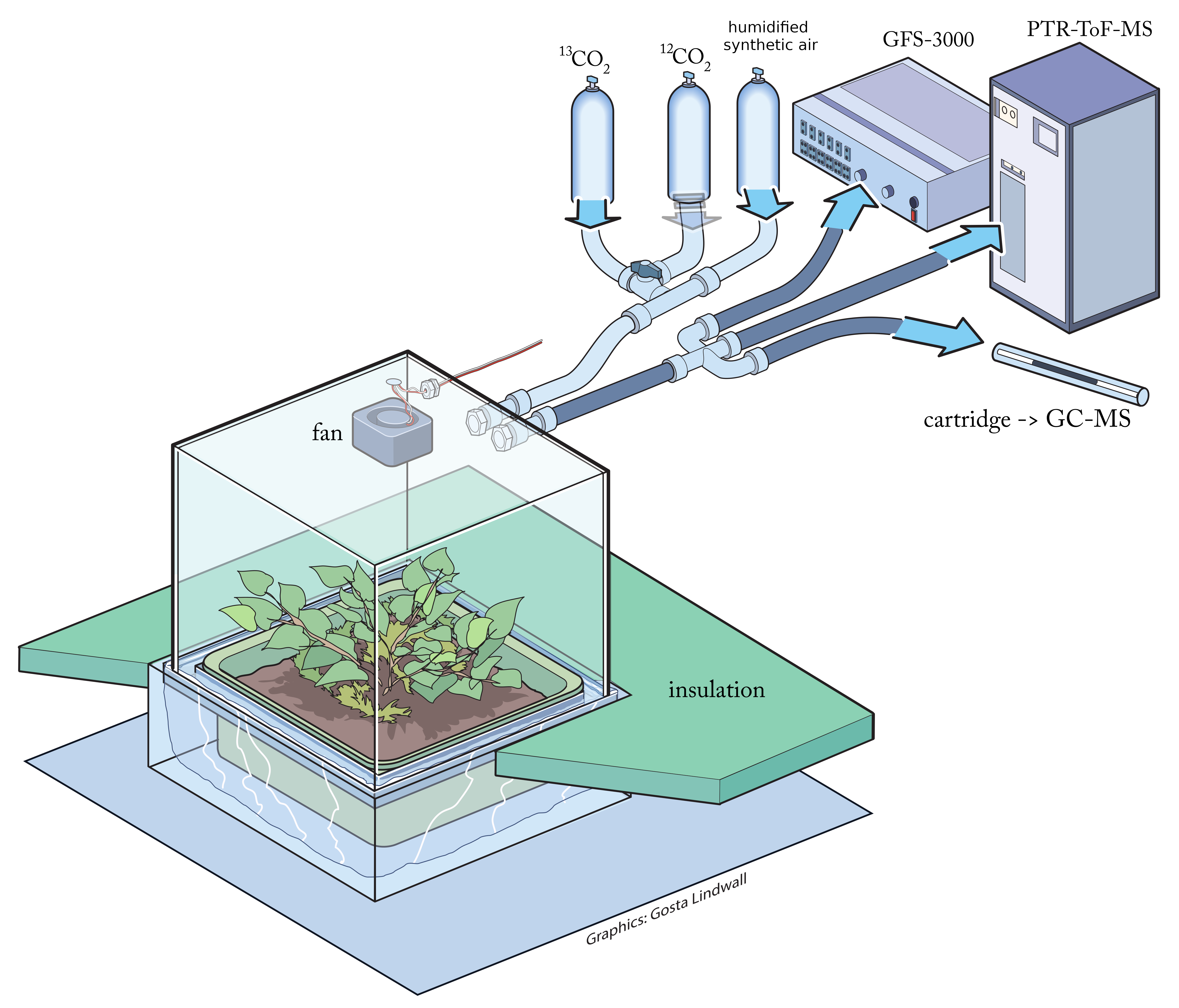
**

**Figure S3. Schematic illustration showing the cuvette system used for the ^13^CO_2_ fumigation exposure and gas analysis of CO_2_ and VOC emissions of subarctic mesocosms.** The whole cuvette system was composed of six cuvettes, which were located inside the sub-chambers of the two phytotron chambers, three for each climate simulation, and run in parallel. Each cuvette consists of an aluminium frame with collar as base (the inner part was 22x22 cm, exactly fitting the PET pot containing the mesocosm), and a transparent polycarbonate lid (thickness of 1.5 mm and a height of 20 cm). To perform gas analysis, the mesocosm was enclosed into the cuvette by placing the PET pot inside the frame and closing the lid into the collar. The tightness of the system was achieved by filling the collar with Milli-Q water. The cuvette was purged continuously at rate of 650 ml min^-1^ with VOC-free synthetic air, mixed with 99% of either ^12^CO_2_ (pre-labeling and control) or ^13^CO_2_ (during labeling) at a concentration of 450 ppmv. Headspace inside the cuvette was kept homogenized by continuously mixing with a fan. The outlet airflow was divided into three PFA tubing lines and directed for gas analyses: i) 100 ml min^-1^ (for 60 min) were passed through glass cartridges filled with Tenax and Carbopack for GC-MS sampling and VOC analysis, ii) ~120 ml min^-1^ to PTR-ToF-MS for online VOC analysis, iii) ~300 ml min^-1^ to GFS-3000 for gas-exchange measurements of CO_2_ and H_2_O. A multiplex system based on six solenoid 3-way valves switched automatically the outlet of each of the six cuvettes every five minutes. All air flows were controlled using mass flow controllers (MKS, Andover, USA), calibrated using a certified mass flow meter (ADM-3000, Agilent Technologies, Palo Alto, USA). To ensure a leak-free procedure, an overflow of approx. 130 ml min^-1^ was maintained. All lines used were 6 mm (OD) PFA tubes and thermally isolated. To keep the different soil and air temperatures set by the phytotron sub-chambers, a Styrofoam sheet was used to insulate the above ground from the below ground parts of the mesocosm.

**
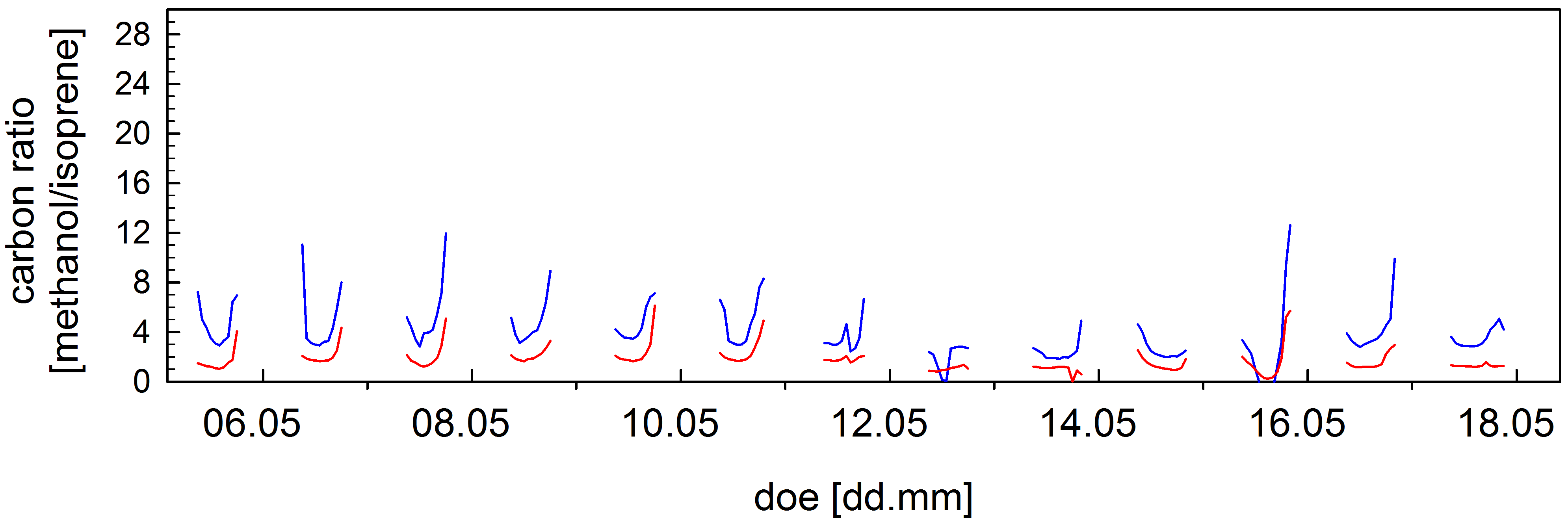
**

**Figure S4. Changes of methanol/isoprene carbon ratios caused by warming climate.** The carbon atom ratios of methanol and isoprene emitted by mixed *Betula* and *Salix* mesocosms growing under control (C, in blue) and warming (W, in red) climate. Data originate from chamber measurements. Data were calculated under reliable, light-dependent isoprene emissions between 9:00-20:00 CET and averaged based at 5 h resolution.

**
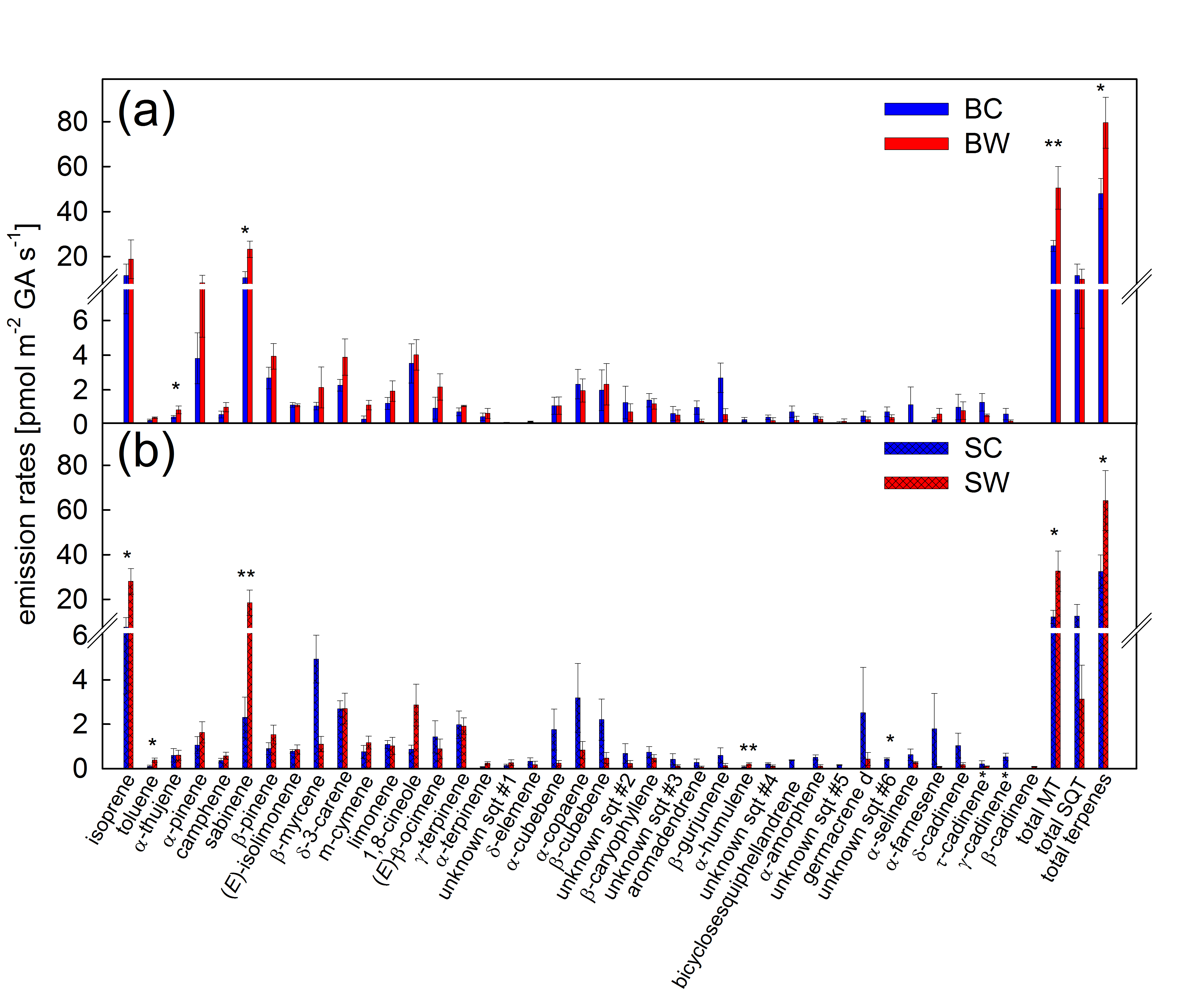
**

**Figure S5. VOC emission rates observed in the morning.** VOC emissions measured by GC-MS in the morning (9:45-10:45 CET) before the ^13^C-labeling from (**A**) ‘*Betula*’ and (**B**) ‘*Salix*’ mesocosms under control (C) and warming (W) climate simulations. Emission rates are given per ground area (GA). Data were collected from individual, cuvette-enclosed mesocosms and analyzed by GC-MS. Statistical comparison of treatment effect within the mesocosm species: **P*<0.05, ***P*<0.01, ****P*<0.001. Means ± se (n = 9). The detailed statistical analysis is given in Supp. Table S3. Abr.: BC, *Betula* under control; BW, *Betula* under warming; SC, *Salix* under control; SW, *Salix* under warming.

**
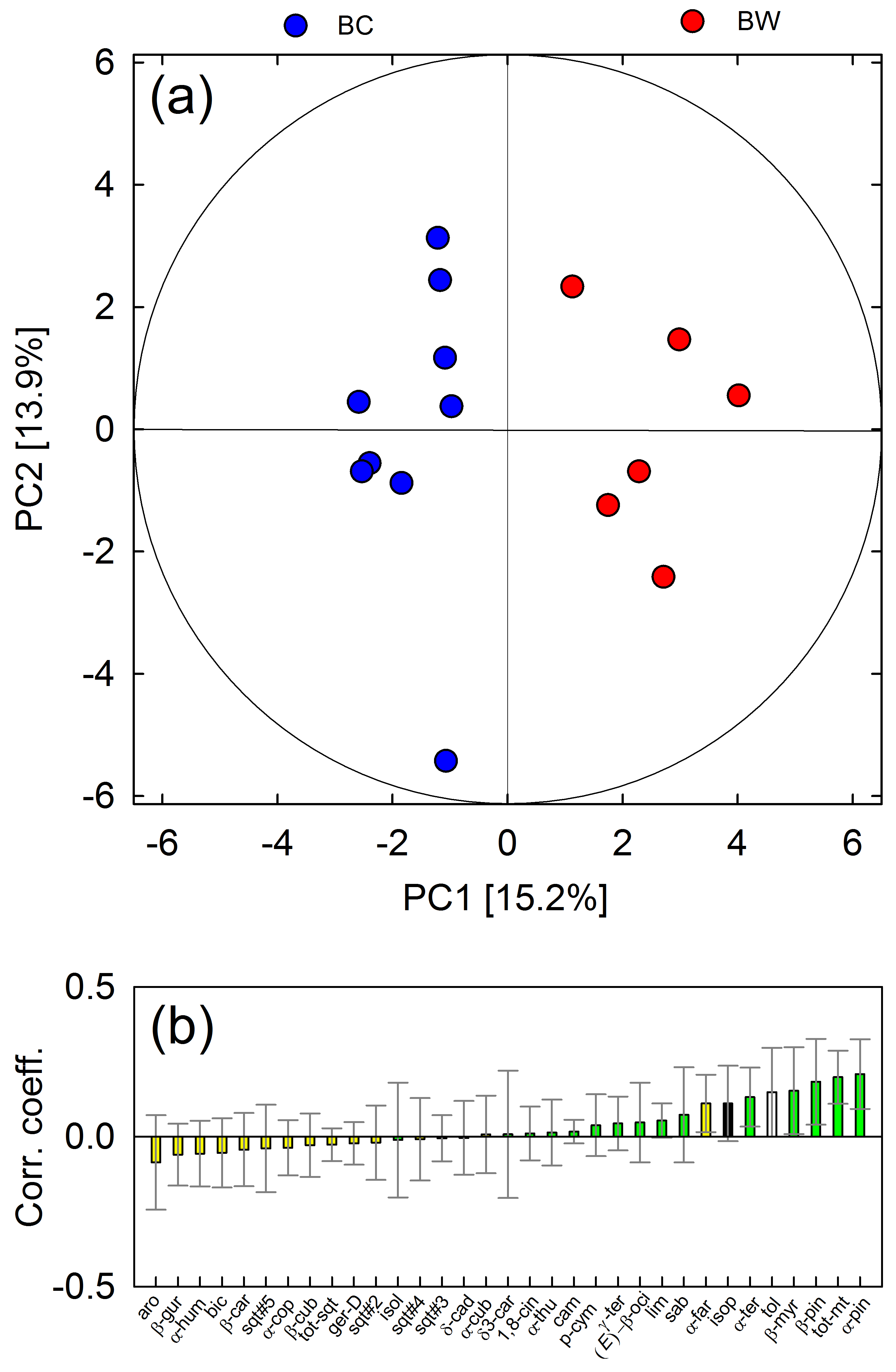
**

**Figure S6. Warming effect on volatile emissions from ‘*Betula’* mesocosm.** Air **s**amples were collected during the last hour of the ^13^C-labeling (15:30-16:30 CET). **a)** Orthogonal partial least square regression (OPLS) score plot of VOC emissions (mesocosm emission rates normalized per ground area, pmol m^-2^ GA s^-1^). The ellipse indicates the model tolerance based on Hotelling’s *T*^2^ and significance level of α = 0.05. **b**) Correlation coefficient plot of OPLS, correlating VOC emissions with treatment (warming) effect. The coefficients are given scaled and centered. The error bars are derived using the jack-knife method. Bars represent the average ± se of 15 mesocosms. OPLS model fitness: *r*^2^ (x)=39.1%, *r*^2^=87%, *q*^2^ (cum)=45% using 1 predictive component. RMSEE (root mean square error of estimation) = 0.19; RMSEcv (root mean square error of cross-validation) = 0.39; *P* < 0.05, CV-ANOVA. Abb.: (*E*)- β-oci , (*E*)- β-ocimene; 1,8-cin, 1,8-cineole; aro, aromadendrene; bic, bicyclosesquiphellandrene; cam, camphene; ger-D, germacrene d; iso, isoprene; p-cym, p-cymene; sab, sabinene; sq#, unknown sqt #; tol, toluene; α-far, α-farnesene; α-pin, α-pinene; α-thu, α-thujene; β-car, β-caryophyllene; β-cub, β-cubebene; β-cad, β-cadinene; β-gur, β-gurjunene; β-myr, β-myrcene; β-pin, β-pinene; α-cop, α-copaene; α-cub, α-cubebene; α-hum, α−humulene; α-ter, α-terpinene; δ-3-car, δ-3-carene; δ-cad, δ-cadinene; δ-ele, δ-elemene; γ-ter, γ-terpinene; τ-cad, τ-cadinene*. *: tentatively identified. PC, predictive component. Color code: (a): control, blue; warming, red; (b) monoterpenes, green; sesquiterpenes, yellow; isoprene, black; benzenoids, white.

**
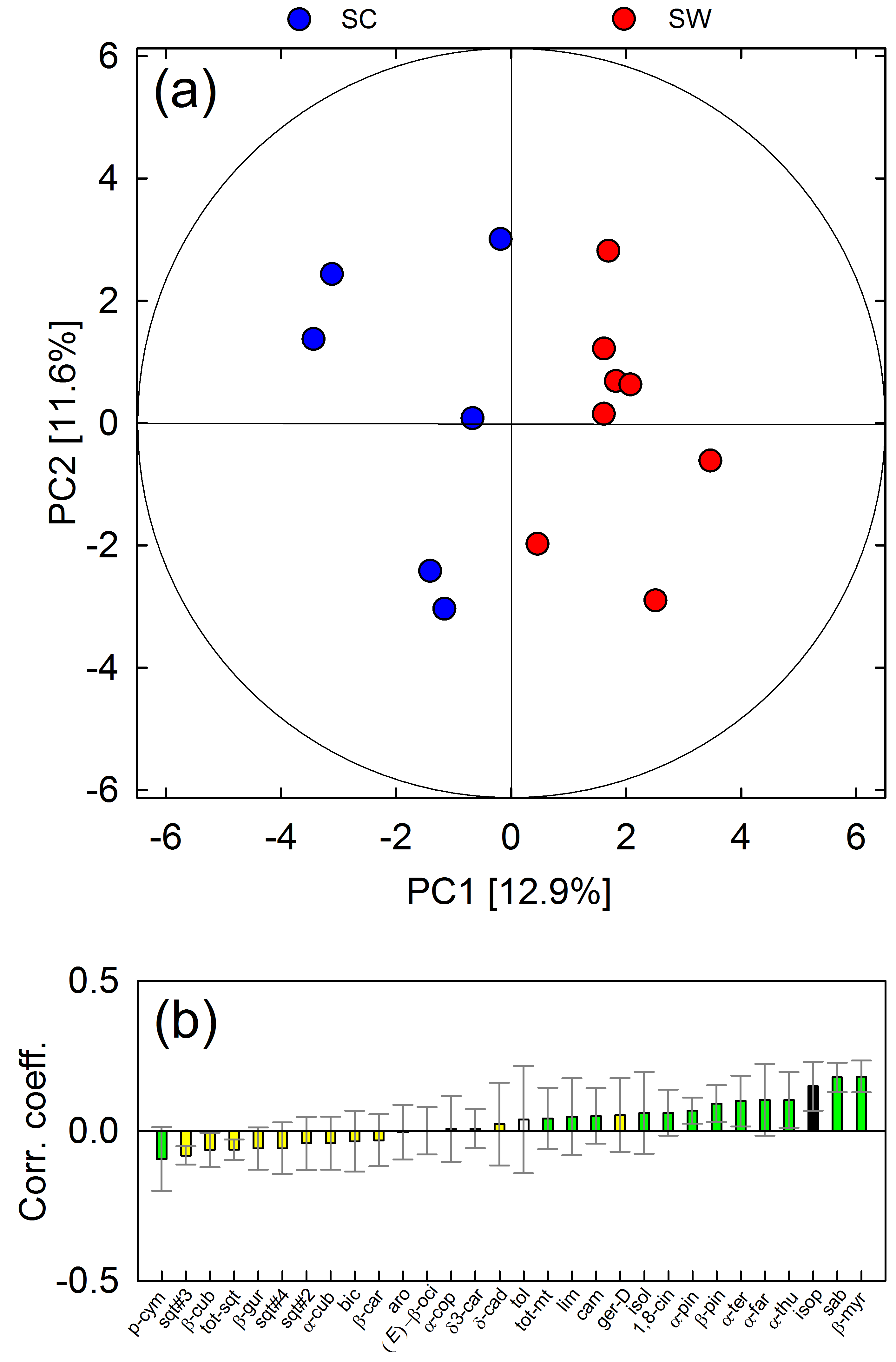
**

**Figure S7. Warming effect on volatile emissions from ‘*Salix*’ mesocosm.** Air **s**amples were collected during the last hour of the ^13^C-labeling (15:30-16:30 CET). **a)** Orthogonal partial least square regression (OPLS) score plot of VOC emissions (mesocosm emission rates normalized per ground area, pmol m^-2^ GA s^-1^). The ellipse indicates the model tolerance based on Hotelling’s *T*^2^ and significance level of α = 0.05. **b**) Correlation coefficient plot of OPLS, correlating VOC emissions with treatment (warming) effect. The coefficients are given scaled and centered. The error bars are derived using the jack-knife method. Bars represent the average ± se of 14 mesocosms. OPLS model fitness: *r*^2^ (x)=38%, *r*^2^=85%, *q*^2^ (cum)=49% using 1 predictive component. RMSEE (root mean square error of estimation) = 0.21; RMSEcv (root mean square error of cross-validation) = 0.35; *P* < 0.05, CV-ANOVA. Abb.: (*E*)- β-oci, (*E*)-β-ocimene; 1,8-cin, 1,8-cineole; aro, aromadendrene; bic, bicyclosesquiphellandrene; cam, camphene; ger-D, germacrene d; iso, isoprene; p-cym, p-cymene; sab, sabinene; sq#, unknown sqt #; tol, toluene; α-amo, α-amorphene; α-far, α-farnesene; α-pin, α-pinene; α-sel, α-selinene; α-thu, α-thujene; β-car, β-caryophyllene; β-cub, β-cubebene; β-cad, β-cadinene; β-gur, β-gurjunene; β-myr, β-myrcene; β-pin, β-pinene; α-cop, α-copaene; α-cub, α-cubebene; α-hum, α−humulene; α-ter, α-terpinene; δ-3-car, δ-3-carene; δ-cad, δ-cadinene; δ-ele, δ-elemene; γ-ter, γ-terpinene. PC, predictive component. Color code: (a): control, blue; warming, red; (b) monoterpenes, green; sesquiterpenes, yellow; isoprene, black; benzenoids, white.


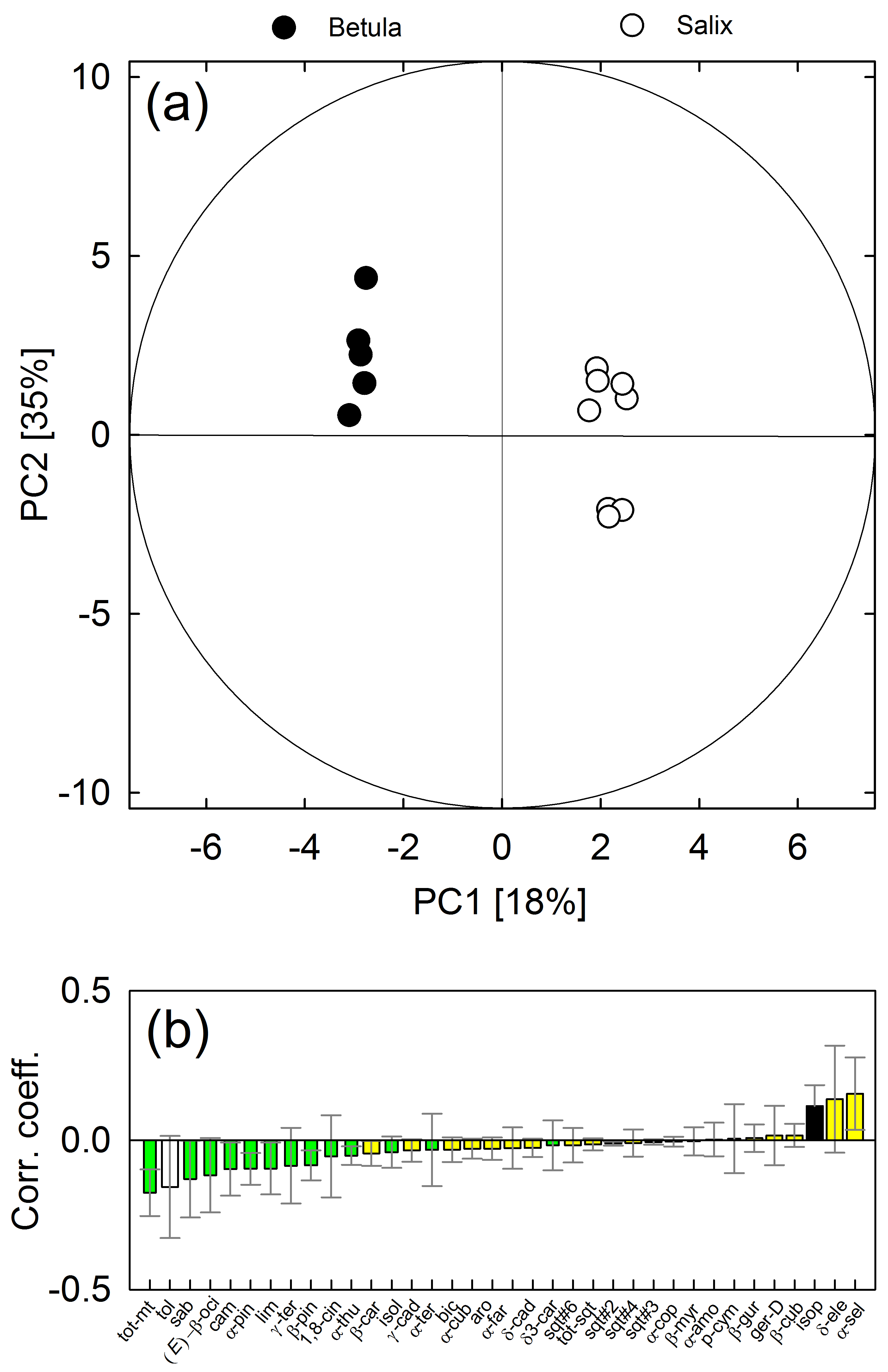


**Figure S8. Volatile differences between Salix and Betula mesocosms under warming treatments.** Air **s**amples were collected during the last hour of the ^13^C-labeling (15:30-16:30 CET). **a)** Orthogonal partial least square regression (OPLS) score plot of VOC emissions (mesocosm emission rates normalized per ground area, pmol m^-2^ GA s^-1^). The ellipse indicates the model tolerance based on Hotelling’s *T*^2^ and significance level of α = 0.05. **b**) Correlation coefficient plot of OPLS, correlating VOC emissions with treatment (warming) effect. The coefficients are given scaled and centered. The error bars are derived using the jack-knife method. Bars represent the average ± se of 13 mesocosms. OPLS model fitness: *r*^2^ (x)=82%, *r*^2^=99%, *q*^2^ (cum)=82% using 1 predictive component. RMSEE (root mean square error of estimation) = 0.05; RMSEcv (root mean square error of cross-validation) = 0.21; *P* < 0.05, CV-ANOVA. Abb.: (*E*)- β-oci , (*E*)- β-ocimene; 1,8-cin, 1,8-cineole; aro, aromadendrene; bic, bicyclosesquiphellandrene; cam, camphene; ger-D, germacrene d; iso, isoprene; p-cym, p-cymene; sab, sabinene; sq#, unknown sesquiterpene #; tol, toluene; α-amo, α-amorphene; α-far, α-farnesene; α-pin, α-pinene; α-sel, α-selinene; α-thu, α-thujene; β-car, β-caryophyllene; β-cub, β-cubebene; β-cad, β-cadinene; β-gur, β-gurjunene; β-myr, β-myrcene; β-pin, β-pinene; α-cop, α-copaene; α-cub, α-cubebene; α-hum, α−humulene; α-ter, α-terpinene; δ-3-car, δ-3-carene; δ-cad, δ-cadinene; δ-ele, δ-elemene; γ-ter, γ-terpinene. PC, predictive component. Color code: (a): control, blue; warming, red; (b) monoterpenes, green; sesquiterpenes, yellow; isoprene, black; benzenoids, white.


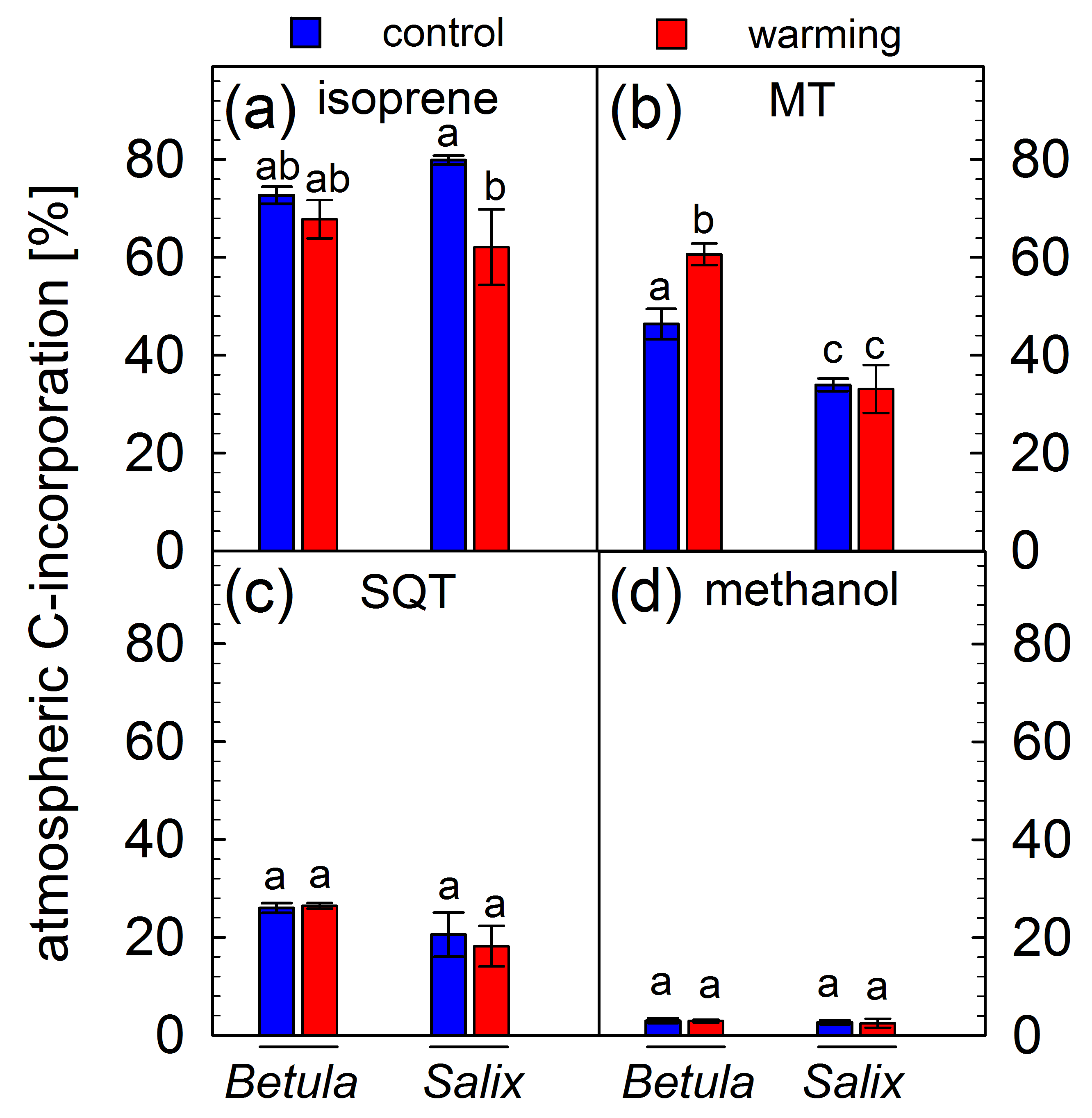


**Figure S9. Incorporation of atmospheric carbon into VOCs.** (**a**) Isoprene, (**b**) monoterpenes, (**c**) sesquiterpenes, (**d**) methanol, emitted by ‘*Betula*’ and ‘*Salix*’ mesocosms under control (in blue) and warming (in red) climate simulation. VOCs were collected from individual, cuvette-enclosed mesocosms and analyzed by PTR-ToF-MS. The atmospheric incorporation of carbon (C) from CO_2_ into VOCs was calculated using ^13^CO_2_ labeling technique as described in methods. Depicted data derive from significant (*P*<0.001, pair *t*-test) ^13^C-labeling of VOC, compared to ^12^CO_2_ control experiments. Different letters denote statistically significant differences (*P*<0.05, ANOVA, and Tukey test method for pairwise multiple comparison procedures). Means ± se. (n=6).


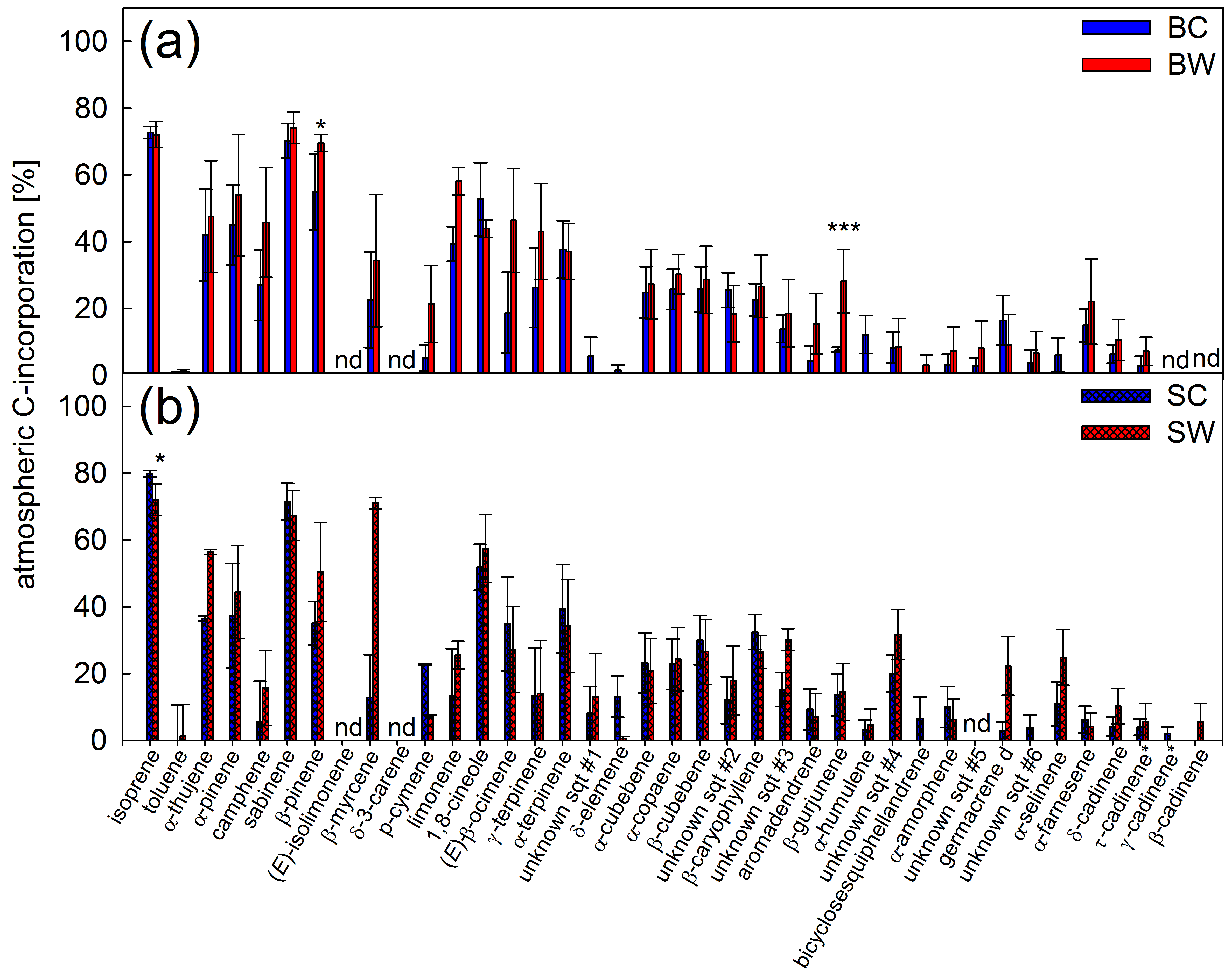


**Figure S10. Incorporation of atmospheric carbon into VOCs**. (**a**) ‘*Betula*’ (B) and (**b**) ‘*Salix*’ (S) mesocosms under control (C, in blue) and warming (W, in red) climate simulation. VOCs were collected from individual, cuvette-enclosed mesocosms and analyzed by GC-MS. The atmospheric incorporation of carbon (C) from CO_2_ into VOCs was calculated using ^13^CO_2_ labeling technique as described in the methods. Statistical significance was tested with ANOVA and the Holm-Sidak method for pairwise multiple comparison procedures. Comparison for factors: C *vs* W within B (***); B *vs* S within W (**). Abbr.: *, *P*<0.05; **, *P*<0.01; ***, *P*<0.001 Means ± se. (n=6); nd, not detectable.

**
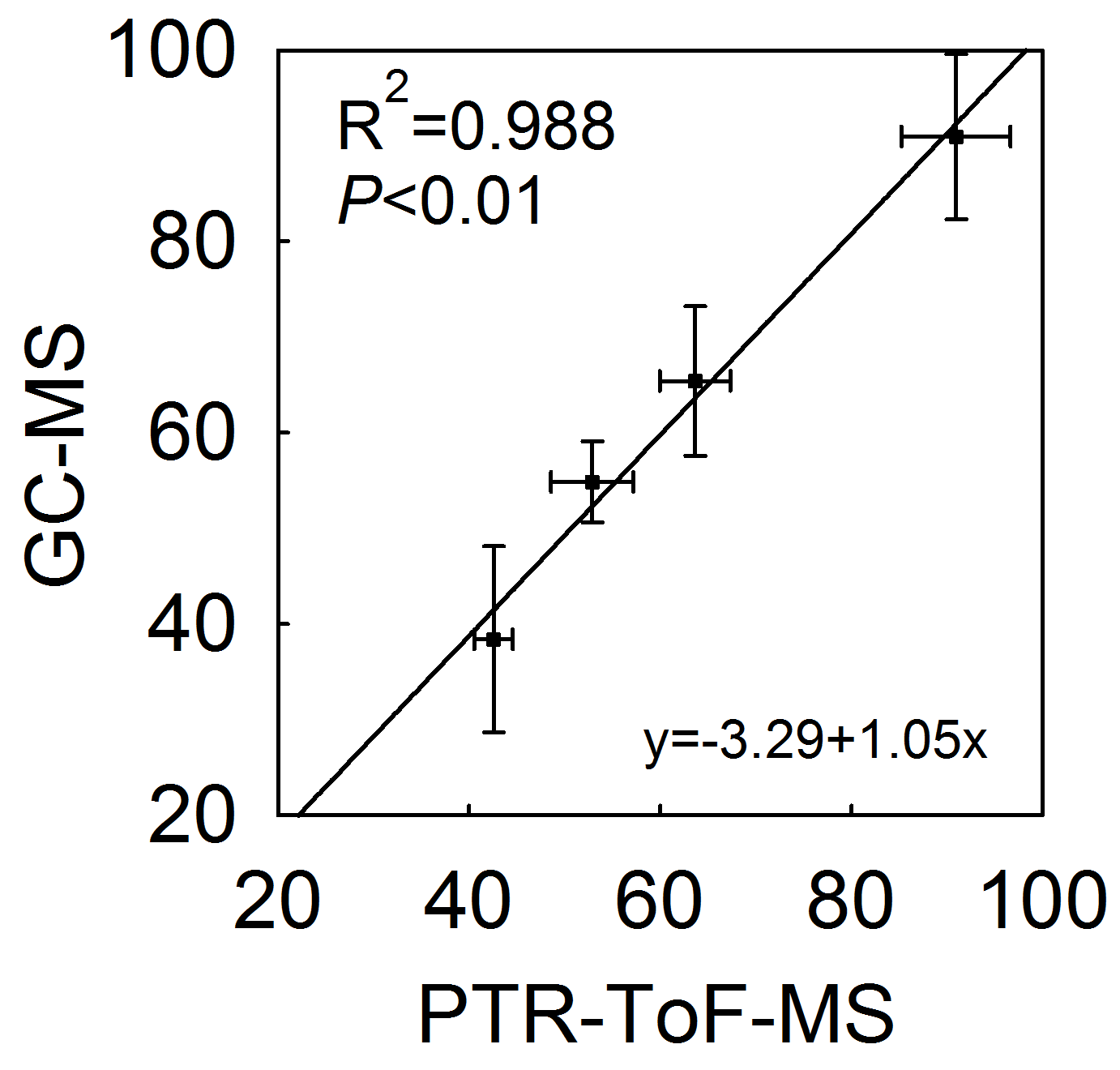
**

**Figure S11. Linear correlation between GC-MS and PTR-ToF-MS data.** Total *de novo* monoterpene biosynthesis (given as percentages). VOCs were collected from individual, cuvette-enclosed mesocosms.


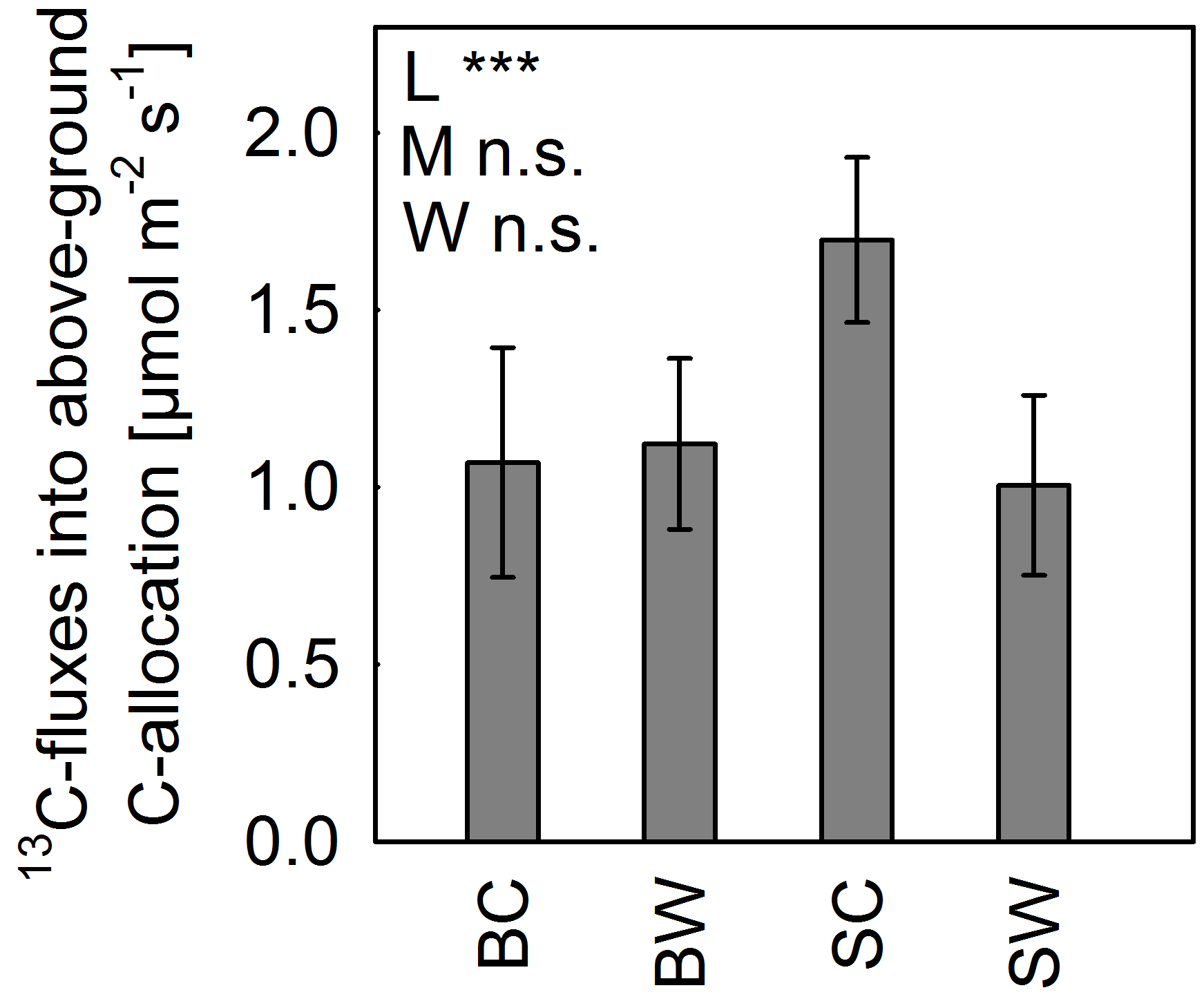


**Figure S12. ^13^C-fluxes from atmospheric ^13^CO_2_ allocated into the aboveground leaf material in the mesocosm**. ‘*Betula*’ (B) and ‘*Salix’* (S), under control (C) and warming (W) treatments. Statistical main effects (L, labeling; M, mesocosm type; W, warming) are reported in panel. Means ± se. (n=6). ***, *P*<0.001.


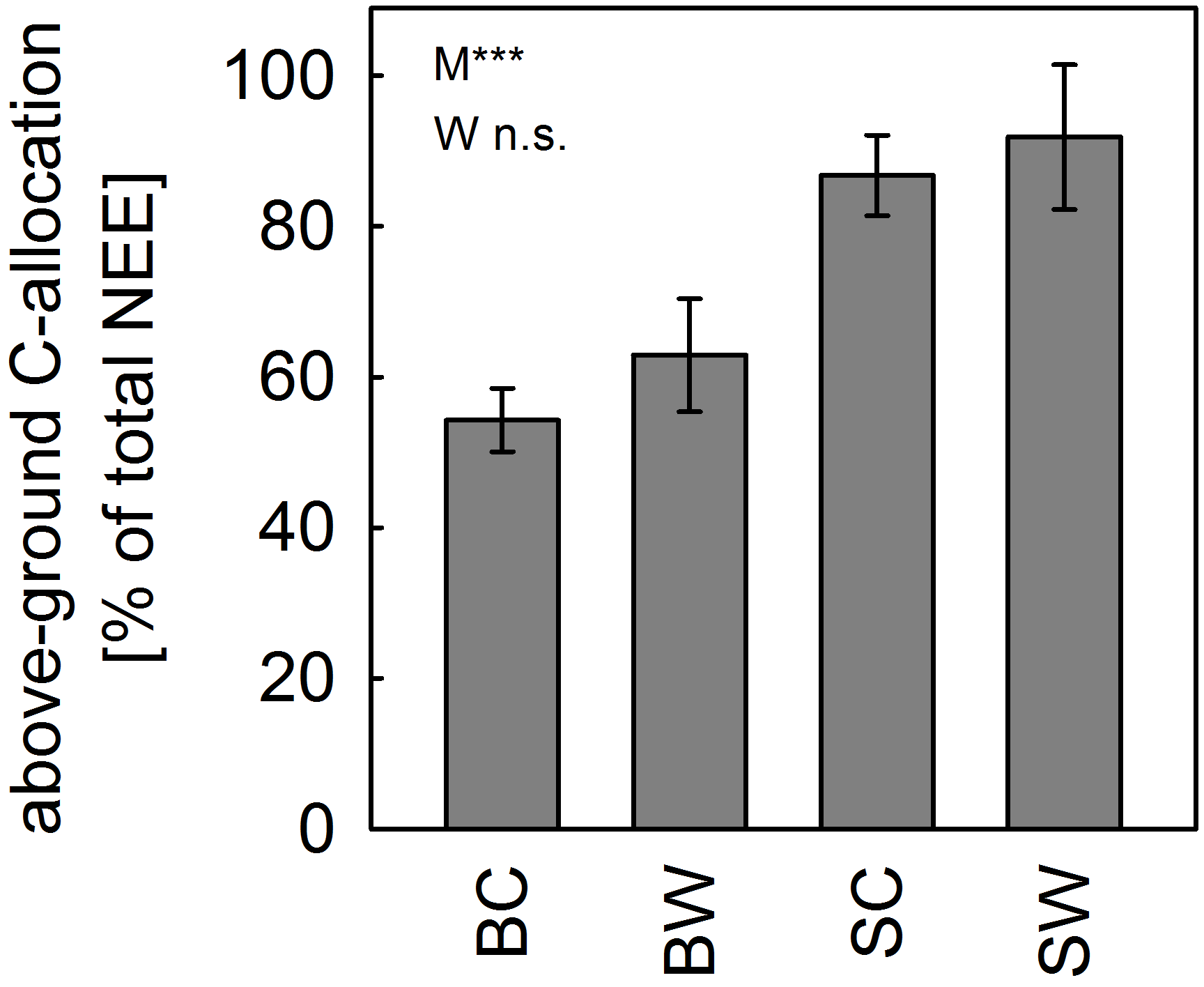


**Figure S13.** **Carbon allocation into the aboveground leaf material, derived by measuring ^13^C-fluxes from atmospheric ^13^CO_2_ to aboveground leaf materials, and to belowground plant material and soil carbon.** ‘*Betula*’ (B) and ‘*Salix’* (S) mesocosms, under control (C) and warming (W) treatments. Statistical main effects (L, labeling; M, mesocosm type; W, warming) are reported in panel. Means ± se. (n=6). ***, *P*<0.001.

**Table S1.** **Plant species composition and biomass.** Plant species composition and biomass (g dw mesocosm^-1^) in mesocosms dominated by ‘*Betula*’ and ‘*Salix*’ under control and warming conditions. Statistical significance of mesocosm types (Meso) and warming treatment (Treat) on plant biomass was tested with 2-way ANOVA. **P*<0.05. Abbr. *f*, foliage; *s*, stem; *f*+*s*, unseparated foliage and stem. Means of n=9 ± se.


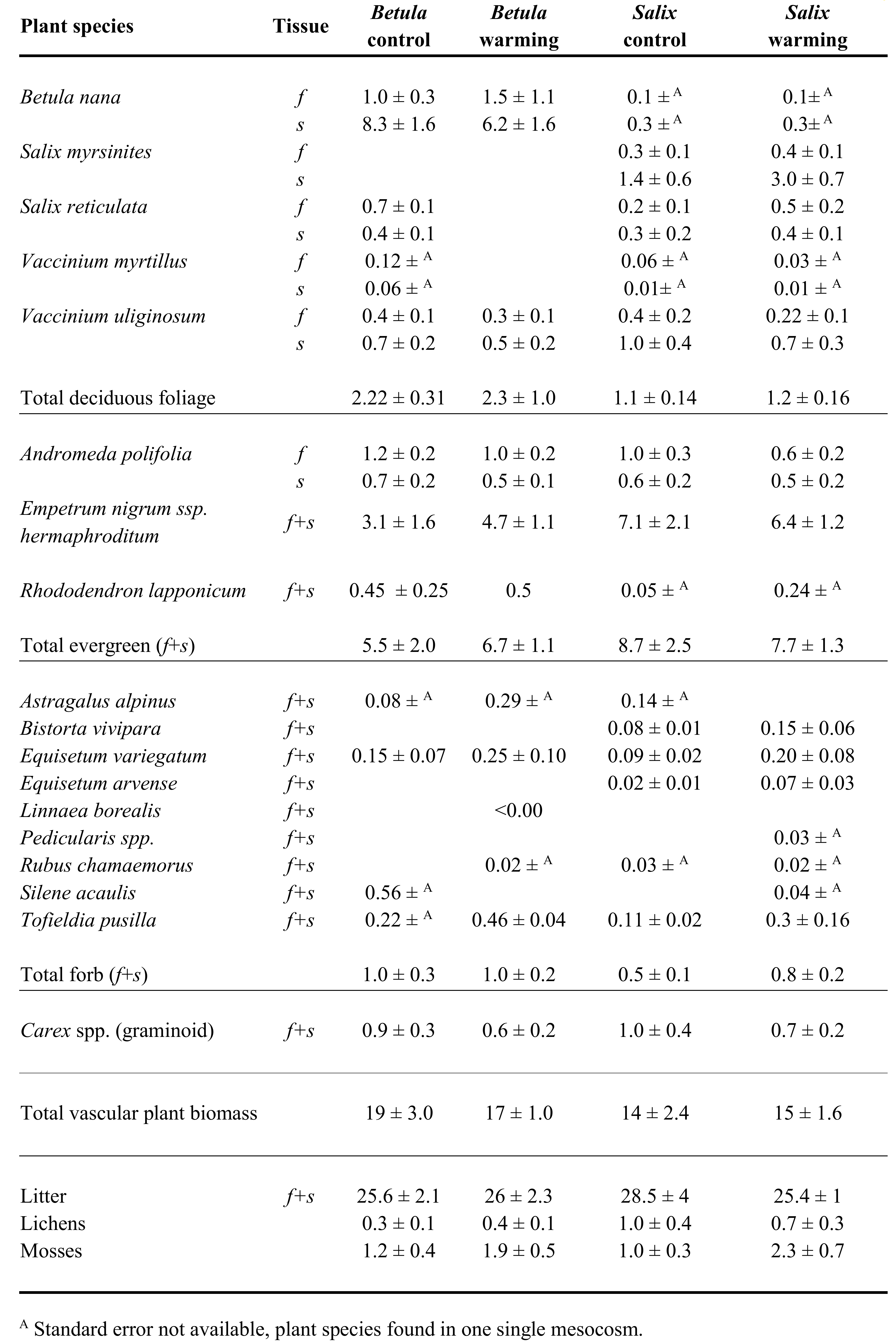


**Table S2**

**Limit of detection (LOD) and protonated masses of the VOCs used to calibrate the PTR-ToF-MS.** The standard mixture (Apel-Riemer Environmental, Denver, CO, USA) was diluted in a range of concentrations of 0-150 ppbv and passed through all the cuvette system (Supp. Figure S3).

**
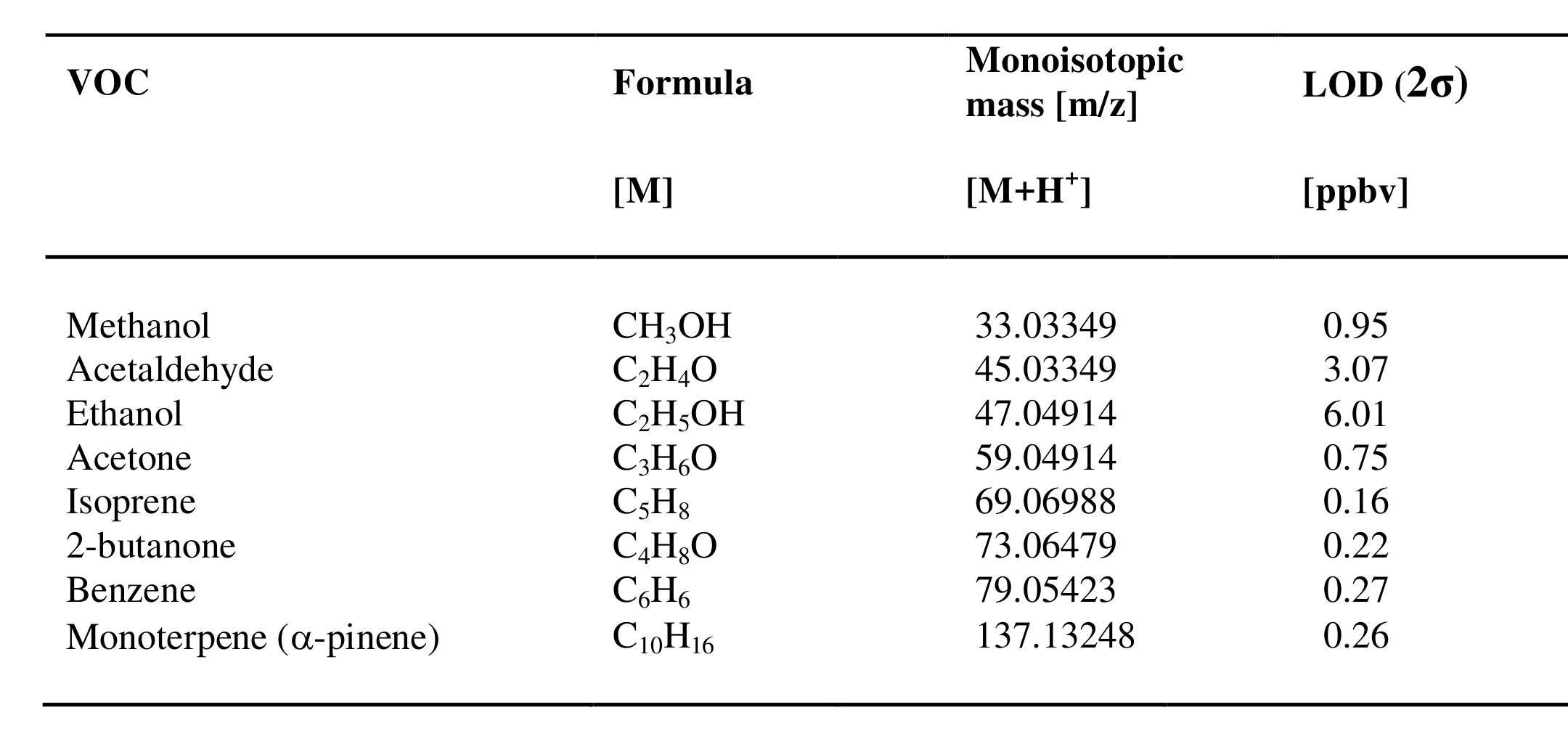
**

**Table S3**

Emission rate normalized to ground area (GA) and chemical identification of VOCs. Main effects of climate, mesocosm type (meso), and sampling times (morning, 09:45-10:45 or afternoon, 15:30-16:30 CET) are indicated with: **P*<0.05, ***P*<0.01, ****P*<0.001. Means ± se (n = 9). B, ‘*Betula*’; S, ‘*Salix*’; C, control; W, warming.


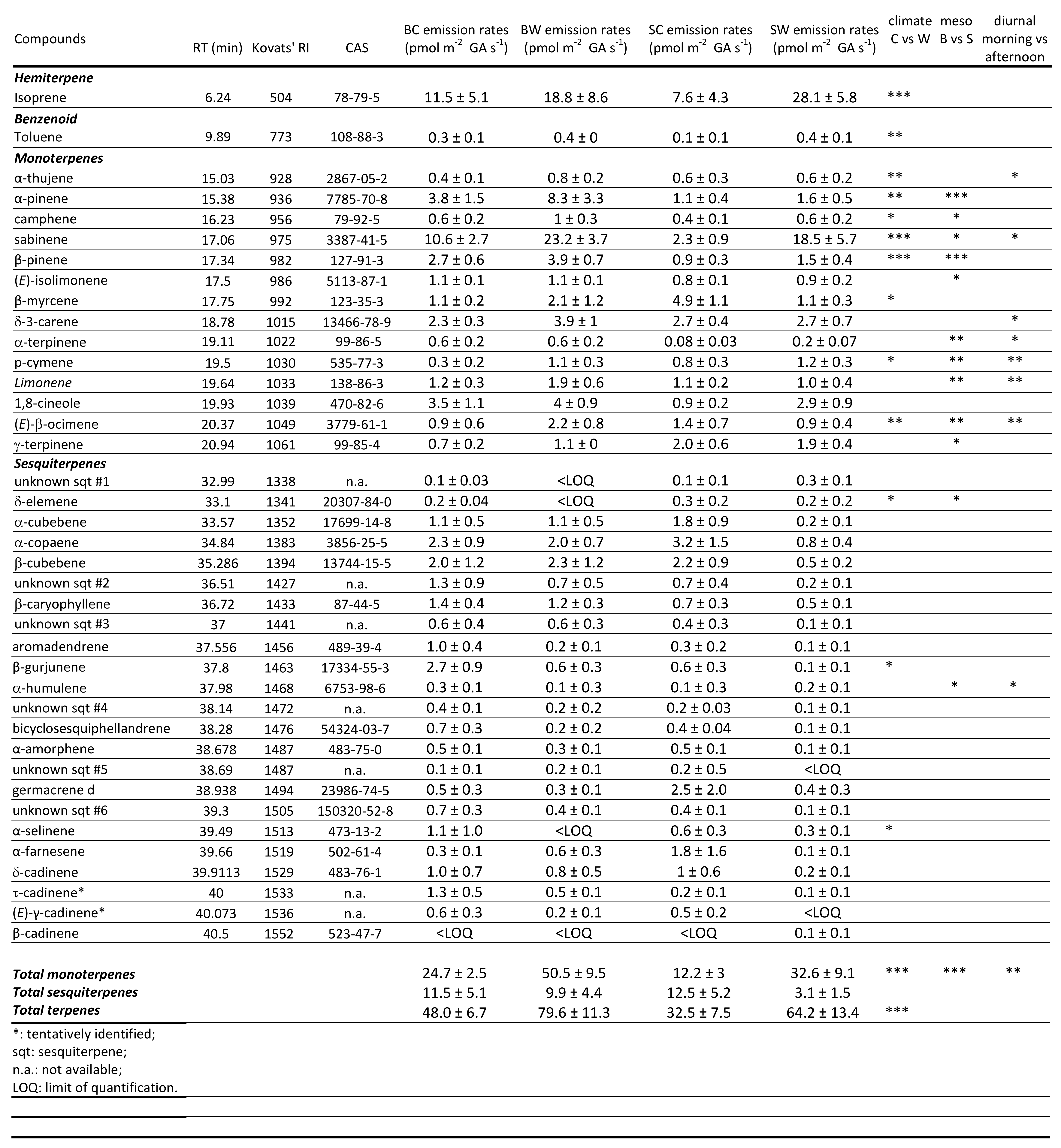

Supplement: Supplementary file 1 — Supinfo [file GCB-26-1908-s001.docx]
